# Hypothesis‐driven investigations of diverse pharmacological targets in two mouse models of autism

**DOI:** 10.1002/aur.2066

**Published:** 2019-01-17

**Authors:** Maya A. Rhine, Jennifer M. Parrott, Maria N. Schultz, Tatiana M. Kazdoba, Jacqueline N. Crawley

**Affiliations:** ^1^ MIND Institute, Department of Psychiatry and Behavioral Sciences University of California Davis School of Medicine Sacramento California 95817

**Keywords:** autism, cognitive, GABA, medicine, mice, preclinical, repetitive, social, Trkb

## Abstract

Autism spectrum disorder is a neurodevelopmental syndrome diagnosed primarily by persistent deficits in social interactions and communication, unusual sensory reactivity, motor stereotypies, repetitive behaviors, and restricted interests. No FDA‐approved medical treatments exist for the diagnostic symptoms of autism. Here we interrogate multiple pharmacological targets in two distinct mouse models that incorporate well‐replicated autism‐relevant behavioral phenotypes. Compounds that modify inhibitory or excitatory neurotransmission were selected to address hypotheses based on previously published biological abnormalities in each model. *Shank3B* is a genetic model of a mutation found in autism and Phelan‐McDermid syndrome, in which deficits in excitatory neurotransmission and synaptic plasticity have been reported. BTBR is an inbred strain model of forms of idiopathic autism in which reduced inhibitory neurotransmission and excessive mTOR signaling have been reported. The GABA‐A receptor agonist gaboxadol significantly reduced repetitive self‐grooming in three independent cohorts of BTBR. The TrkB receptor agonist 7,8‐DHF improved spatial learning in *Shank3B* mice, and reversed aspects of social deficits in BTBR. CX546, a positive allosteric modulator of the glutamatergic AMPA receptor, and d‐cycloserine, a partial agonist of the glycine site on the glutamatergic NMDA receptor, did not rescue aberrant behaviors in *Shank3B* mice. The mTOR inhibitor rapamycin did not ameliorate social deficits or repetitive behavior in BTBR mice. Comparison of positive and negative pharmacological outcomes, on multiple phenotypes, evaluated for replicability across independent cohorts, enhances the translational value of mouse models of autism for therapeutic discovery. GABA agonists present opportunities for personalized interventions to treat components of autism spectrum disorder. ***Autism Res*** 2019, 12: 401–421 © 2019 The Authors. *Autism Research published by International Society for Autism Research* published by Wiley Periodicals, Inc.

**Lay Summary:**

Many of the risk genes for autism impair synapses, the connections between nerve cells in the brain. A drug that reverses the synaptic effects of a mutation could offer a precision therapy. Combining pharmacological and behavioral therapies could reduce symptoms and improve the quality of life for people with autism. Here we report reductions in repetitive behavior by a GABA‐A receptor agonist, gaboxadol, and improvements in social and cognitive behaviors by a TrkB receptor agonist, in mouse models of autism.

## Introduction

Growing knowledge about genetic mutations and copy number variants in autism spectrum disorder provides insights into potential therapeutic targets [Muhle, Reed, Stratigos, & Veenstra‐Vanderweele, 2018]. While effective behavioral interventions remain the current standard of care [Lord & Jones, 2013; Rogers et al., 2014; Cidav et al., 2017], the combination of behavior therapy with pharmacological intervention could synergistically improve symptoms, trajectory, and quality of life. At present, no medical treatments have been approved by the U.S. Food and Drug Administration for the diagnostic symptoms of autism, specifically the core features of social deficits and repetitive behaviors. As many of the risk genes for autism impact the functions of synapses in the brain [Bourgeron, 2009; Baudouin et al., 2012; De Rubeis, He, Goldberg, et al., 2014; O'Roak et al., 2014; Iossifov, O'Roak, Sanders, et al., 2014; Pinto et al., 2014; Washbourne, 2015; Sanders et al., 2015; de la Torre‐Ubieta, Won, Stein, & Geschwind, 2016; Yuen, Merico, Bookman, et al., 2017], drugs that ameliorate synaptic dysfunctions could conceivably offer precision therapeutics for cases of autism with identified synaptic etiologies. Animal models that incorporate mutations in risk genes for autism, and display behaviors with relevance to the diagnostic symptoms of autism, can approximate but never fully recapitulate all components of the human syndrome. Mutant mouse models offer translational tools to investigate potential pharmacological targets for personalized treatments of the core symptoms of autism [Spooren, Lindemann, Ghosh, & Santarelli, 2012; Kazdoba et al., 2016a; Chadman, 2017].

To advance preclinical discovery of effective therapeutics for the diagnostic symptoms of autism, we evaluated five diverse classes of compounds in two mouse models of autism with divergent imbalances in excitatory/inhibitory neurotransmission. *Shank3* mutations are strongly implicated as a monogenic cause of autism, and are central to the chromosome 22q13.3 deletion which causes Phelan‐McDermid syndrome, a neurodevelopmental disorder presenting with intellectual impairments, seizures, and autism [Durand et al., 2007; Betancur & Buxbaum, 2013; Monteiro & Feng, 2017]. The Shank family of postsynaptic cytoskeletal scaffolding proteins regulates the development and functioning of glutamatergic synapses, as shown in several lines of *Shank3* mutant mice that were generated with different mutations within the *Shank3* gene. Dependent on the specific mutation, and the brain region containing the mutation in cases of conditional knockouts, *Shank3* mutant mice displayed reduced frequency and amplitude of miniature excitatory postsynaptic currents [Bozdagi et al., 2010; Peca et al., 2011], reduced glutamatergic transmission and impaired long‐term potentiation in the hippocampus [Wang et al., 2011; Yang et al., 2012; Jaramillo et al., 2017], reductions in the NMDA/AMPA ratio in hippocampal CA1, prefrontal cortex, and striatal medium spiny neurons [Kouser et al., 2013; Duffney et al., 2015; Jaramillo et al., 2016;. Lee et al., 2015; Wang et al., 2017], and resistance to pentyletetrazole‐induced seizures [Dhamne et al., 2017], indicative of reduced excitatory physiology. *Shank3B* null mutant mice phenocopy the two diagnostic symptom categories of autism, displaying low scores on social assays and high levels of repetitive self‐grooming [Peca et al., 2011; Chang et al., 2016; Wang et al., 2017; Dhamne et al., 2017]. Shank3 has been implicated in synaptic plasticity, and in the functions of NMDA, AMPA and mGluR5 receptors, through mechanisms including dendritic actin stabilization [de Bartolomeis, Latte, Tomasetti, & Iasevoli, 2014; Duffney et al., 2015; Zhou et al., 2016; Mei et al., 2016; Verpelli et al., 2011; Lovinger, 2017; Vicidomini et al., 2017]. On the basis of these reports, compounds that increase excitatory neurotransmission promote synapse formation were selected for testing on behavioral phenotypes in *Shank3B* mice.

BTBR T(+)Itpr3(tf)/J (BTBR) is an inbred strain of mice that displays well‐replicated social deficits and high levels of repetitive behavior [Bolivar, Walters, & Phoenix, 2007; Moy et al., 2007; Yang, Zhodzishsky, & Crawley, 2007; McFarlane et al., 2008; Pobbe et al., 2010; Pearson et al., 2011, 2012; Gould et al., 2012; Silverman et al., 2012, 2015; Burket, Benson, Tang, & Deutsch, 2014; Yoshimura et al., 2017; Bove et al., 2018]. Abnormalities in genetic loci and signaling pathways have been documented in BTBR mice, including levels of brain derived neurotrophic factor (BDNF), corticosterone, kynurenine 3‐hydroxylase and extracellular signal‐regulated kinase (ERK) [McFarlane et al., 2008; Silverman et al., 2010; Frye & Llaneza, 2010; Stephenson et al., 2011; Jones‐Davis et al., 2013; Scattoni, Martire, Cartocci, Ferrante, & Ricceri, 2013; Seese, Maske, Lynch, & Gall, 2014; Daimon et al., 2015]. On the basis of these reports, compounds that reduce excitatory neurotransmission and promote synapse formation were selected for testing on behavioral phenotypes in BTBR mice. Since specific risk gene mutations associated with autism have not yet been identified in BTBR, this inbred strain offers a model of idiopathic autism, paralleling the large proportion of individuals with autism for whom no genetic and/or environmental causes are apparent.

## Methods

### *Mice*

All mice were purchased from The Jackson Laboratory (JAX) in Bar Harbor, Maine, and bred at the University of California Davis in Sacramento. Mice were housed in ventilated Tecniplast cages in an AAALAC approved temperature‐controlled vivarium on a 12:12 circadian cycle with lights on at 7 AM. All husbandry, breeding, behavioral testing, and drug treatment procedures were approved by the University of California Davis Institutional Animal Care and Use Committee, and were conducted in compliance with the NIH Guide for the Care and Use of Laboratory Animals.

Breeding pairs of C57BL/6 J mice (B6, JAX #000664) and BTBR T+ Itpr3^tf^/J (BTBR, JAX #002282) were purchased from JAX and bred in trios to generate full Ns of independent cohorts of experimental subjects for each drug study. B6 and BTBR offspring were group housed by sex and strain. Breeding pairs of *Shank3B* heterozygotes (Shank^*3tm2Gfng*^/J, JAX # 017688, with a targeted deletion in the PDZ domain of the *Shank3* gene, originally generated by Guoping Feng) were purchased from JAX. Our previous testing of *Shank3B* mice revealed strong behavioral phenotypes primarily in the null mutant genotype [Dhamne et al., 2017]. Heterozygotes were therefore used primarily for breeding the WT and homozygous mutants used as subject mice. Heterozygotes of the first and subsequent generations were used as breeding pairs to yield full Ns of separate cohorts of experimental subjects for drug studies. *Shank3B* null mutants (−/−), heterozygotes (+/−) and wildtypes (+/+) were housed by sex and usually by litter, with +/+, +/− and −/− genotypes included in most cages.

### *Methodological Considerations*

Behavioral assays were conducted at 6–16 weeks of age, unless otherwise specified. Behavioral testing was conducted during the light phase of the circadian cycle, between 8:30 AM and 5:30 PM. Mice in their home cages were habituated to the testing room for 1 hr before the start of the behavioral test. Each independently bred cohort of B6 and BTBR mice, and of WT and *Shank3B* null mutant mice, was used for one pharmacological class only. The series of treatments and tests were conducted in the same sets of mice, generally following the sequence of (a) open field exploratory activity, (b) three‐chambered social approach, (c) spontaneous self‐grooming in an empty cage, (d) male–female reciprocal social interactions. In some cohorts, open field activity was evaluated later in the sequence, with similar results obtained. In the initial gaboxadol BTBR and B6 cohorts, the sequence of testing began with elevated plus‐maze and light↔dark transitions anxiety‐related tests. In the TrkB *Shank3B* cohort, water maze was conducted last. Our previous studies with B6, BTBR, and *Shank3*B had revealed similar scores in males and females on the behavioral assays used in the present studies [Yang et al., 2007; McFarlane et al., 2008; Silverman et al., 2012, 2015; Kazdoba et al., 2016a; Dhamne et al., 2017], therefore both males and females were approximately equally represented in the Ns for each cohort, with the exception of male–female reciprocal social interactions in which only the males were tested. Breeding were designed to yield N = 12–15 per treatment group, to provide sufficient power to detect drug effects, as confirmed in previous studies [Silverman et al., 2012, 2015; Silverman, Oliver, Karras, Gastrell, & Crawley, 2013; Kazdoba et al., 2016b; Stoppel et al., 2018]. Inconsistent Ns within a cohort across a testing sequence were the result of occasional cases of equipment failures, injection errors, and unexplained deaths. Video recording was conducted with digital closed‐circuit television cameras (Panasonic, Secaucus, NJ). Investigators conducting the testing and scoring of videos remained uninformed of treatment condition and genotype through the use of coded mouse identification numbers, numerically coded videos, and coding of drug and vehicle vials by another investigator. Each subject mouse was weighed on the morning of each drug treatment, to calculate dose by body weight. All drugs and doses were within ranges previously published as producing no adverse health effects, as described below. Surfaces of each testing chamber were cleaned with 70% ethanol between each subject mouse.

### *Behavioral Testing Procedures*

*Elevated plus‐maze* testing for anxiety‐related behavior was conducted as previously described [Silverman et al., 2015; Kazdoba et al., 2016b]. The automated apparatus (Med Associates, St. Albans City, VT) consisted of a black plastic T‐shaped runway, 1 m above the floor, in which two of the arms have no walls (open arms) and two arms have black walls (closed arms). Photocells recorded transitions between arms. Software calculated time spent in open arms, time spent in closed arms, and transitions between arms. The testing room was illuminated at 300 lux to increase the conflict between exploring a novel environment versus remaining in the closed arms. The 5 min test session began with placing the subject mouse on the central square with forepaws in the junction of the maze and hind paws in an open arm.

*Light↔dark transitions* testing for anxiety‐related behavior was conducted as previously described [Silverman et al., 2010]. The automated apparatus (Research Services Branch, NIMH, Bethesda, MD) consisted of a rectangular plastic box divided into two compartments, one with clear walls and no overhead covering (light chamber) and the other with opaque black walls and an opaque black cover (dark chamber). The testing room was illuminated at 300 lux to increase the conflict between exploring a novel environment and remaining in the dark enclosed area. Photocells across the opening between compartments detected crossings between the light and dark areas. Software calculated time in each chamber and number of transitions between chambers. The 10‐min test session began with placing the subject mouse in the center of the light chamber.

Elevated plus‐maze and light↔dark transitions testing was conducted as a component of the gaboxadol treatment, consistent with previous reports on this compound, but not for other treatments in the current studies. Anxiety‐related tests were conducted first in the gaboxadol testing sequence, consistent with previous literature suggesting that anxiety‐related tests are best conducted in naïve mice (Holmes & Rodgers, 1998).

*Open field* exploratory locomotor activity was conducted as previously described [Silverman et al., 2010, 2015; Kazdoba et al., 2016b; Dhamne et al., 2017]. The automated apparatus consisted of four photocell‐equipped open field chambers and VersaMax Animal Activity Monitoring System (AccuScan Instruments, Columbus, OH). Photocell arrays in the x, y, and raised z locations detected horizontal and vertical movement. Software calculated total distance, horizontal activity, vertical activity, and center time, in 5 min time bins and as totals for the session. The testing room was dimly illuminated at 30 lux. Session duration was 30 min, with the exception of 60 min for the TrkB treatment in B6 and BTBR mice. The test session began with placing the subject mouse in the center of the open field.

*Three‐chambered social approach* was conducted as previously described [Yang, Silverman, & Crawley, 2011; Silverman et al., 2012, 2015; Bales et al., 2014; Brielmaier et al., 2014; Kazdoba et al., 2016b; Dhamne et al., 2017]. The three‐chambered apparatus (Tap Plastics, Sacramento, CA) consisted of a rectangular white nonreflecting plastic box, divided into three equal chambers with small openings in the walls between chambers. The testing room was dimly illuminated at 30 lux. Behaviors were tracked with infrared cameras using the Noldus Ethovision XT9 system. During Phase 1, subject mice were allowed to explore the center chamber with both side chamber doors closed for a 5 or 10‐min habituation session. During Phase 2, the doors to the side chambers were raised and subject mice were allowed to explore all three empty chambers for 10 min. The subject was then briefly confined to the center chamber between closed doors while a clean novel object (an inverted stainless steel wire pencil cup, Galaxy Cup, Kitchen Plus, http://www.kitchen-plus.com or amazon.com, painted white to eliminate glare for video tracking purposes) was placed in one side chamber. A second identical novel wire cup containing a novel stimulus mouse of the same sex and approximate age was placed in the other side chamber. Stimulus mice were an inactive strain, 129S1/SvImJ (JAX catalogue # 002448), previously habituated to the wire cup during a 30‐min habituation session on each of the 2 days prior to testing, to ensure that the stimulus mouse sat quietly inside the wire cup during the test session, allowing all social approach to be initiated by the subject mouse. During Phase 3, doors to the side chambers were raised, and the subject was allowed to explore all three chambers for 10 min. Noldus software automatically recorded time in each chamber, entries into each chamber, and time spent with the subject's nose pointed toward the target and within 2 cm of the perimeter of the wire cage. Observer scoring of sniff time was also conducted in some cases.

*Repetitive self‐grooming* was evaluated as previously described [Yang et al., 2012; Kazdoba et al. 2016b; Dhamne et al., 2017]. Cage changing was scheduled for at least 2 days before testing. Each subject mouse was individually placed in an empty standard mouse cage covered with a filter top lid. A new clean cage was used for each subject mouse. The testing room was dimly illuminated at 20–40 lux. Each subject mouse was recorded during a 10‐min habituation session and a sequential 10‐min testing session, with the camera located above the cage. Coded DVD videos were subsequently scored using a stopwatch, for number of seconds spent engaged in self‐grooming and number of grooming bouts during the 10‐min testing session. The observer noted any motor stereotypies or other unusual spontaneous behaviors, however none were observed in any of the present studies. Grooming included licking, nibbling, and scratching of fur, limbs and tail, usually in the normal sequence that appears during standard self‐grooming in mice.

*Male–female reciprocal social interactions* were evaluated as previously described [Yang et al., 2012; Silverman et al., 2012; Kazdoba et al., 2016b; Dhamne et al., 2017]. The testing apparatus consisted of an environmental chamber (ENV‐018 V; Med Associations, St. Albans, VT), with interior walls covered with convoluted foam sheets (Uline, Pleasant Prairie, WI) for soundproofing, and under dim red lighting conditions at 10 lux. Testing was conducted in a clean mouse cage or Noldus Phenotyper 3000 arena within the sound‐attenuated environmental chamber. An ultrasonic microphone (Avisoft UltraSoundGate condenser microphone CM15; Avisoft Bioacoustics, Glienicke, Germany) was mounted above the cage, and a side‐mounted video camera was focused on the cage. Immediately prior to the test session, each male subject mouse and each female partner mouse was isolated for 1 hr in a clean cage with bedding and without food or water. Partner females were unfamiliar B6 in estrus or proestrus. To induce estrus, partner females were exposed to male scents in cage bedding and urine for three consecutive days prior to the test session, and were confirmed by the appearance of an open vagina surrounded by pink or reddish pink tissue. After the 1 hr isolation period, one subject male and one target female were placed in a clean cage inside the environmental chamber. The 5‐min session was recorded by the video camera and microphone. At the end of the 5‐min test session, each mouse was returned to its isolation cage until all cage mates had been tested. A different clean test cage was used for each subject mouse.

Frequency and duration of each social parameter detected in the male subject mouse were subsequently scored by investigators from coded DVD videos using Noldus Observer event recording software (Noldus Information Technology, Leesburg, VA). Parameters scored included following, nose‐to‐nose sniffing, nose‐to‐anogenital sniffing, sniffing of other body regions, mounting, self‐grooming, and arena exploration. Ultrasonic vocalizations were scored for number of calls using Lab Pro Sound Analysis Software (Avisoft Bioacoustics, Glienicke, Germany). Based on literature showing that calls emitted during male–female social interactions are emitted almost entirely by the male [White, Prasad, Barfield, & Nyby, 1998; Wang, Liang, Burgdorf, Wess, & Yeomans, 2008], number of calls detected was considered to be number of calls emitted by the male subject mice.

#### *Morris water maze*

Mice were trained to locate a hidden platform in a pool of water using distal spatial cues as previously described [Yang et al., 2012; Leach & Crawley, 2018]. The pool was 122 cm diameter, filled with warm water (~22°C), with white nontoxic paint added to make the water opaque. Following training sessions and probe trials, mice were gently dried using towels and a heat lamp to prevent hypothermia. On each training day, four consecutive trials were administered, with a maximum duration of 60 sec per trial. Each mouse was placed into the pool at a starting quadrant location which rotated through the training sessions. Mice unable to locate the platform during initial training trials were guided to the platform and allowed 15 sec rest on the platform before the next trial. Probe trials, in which the hidden platform was removed, were performed at 5 hr after the last training trial to confirm learning using distal cues, and at 24 hr after the last training trial to evaluate long‐term memory. A Noldus EthoVision XT videotracking system recorded the training sessions and probe trials and analyzed components of performance.

### *Drug Preparation and Administration*

Drug treatments and doses are described below. Doses of each compound were previously pubished, as described below, and safety confirmed. At least 2 days intervened between testing days.

The GABA‐A agonist gaboxadol hydrochloride (gaboxadol or THIP, Sigma‐Aldrich, St. Louis, MO) was given acutely, 30 min before the start of the behavioral assay on each testing day. Gaboxadol was freshly prepared on each morning of testing in a vehicle of sterile 0.9% USP grade sodium chloride (Hospira Inc., Lake Forest, IL). Mice were treated intraperitoneally (i.p.) with vehicle, 3 mg/kg, or 5 mg/kg, injected in a volume of 10 ml/kg. Separate groups of mice were used for each drug dose or vehicle treatment with one exception. To reduce the total numbers of mice used, a crossover drug design was employed in one gaboxadol cohort, in which half the mice first received vehicle and subsequently received drug for the entire testing sequence, and the other half first received drug and then subsequently received vehicle, for the entire testing sequence. Comparison of data from the single use and crossover use of mice indicated no differences in drug responses in the two designs. Doses were chosen from previous publications on behavioral actions of gaboxadol in mice [Corbett, Fielding, Cornfeldt, & Dunn, 1991; Elfline, Branda, Babich, & Quock, 2004; Peixoto et al., 2005; Saarelainen et al., 2008; Olmos‐Serrano, Corbin, & Burns, 2011; Ramaker, Ford, Phillips, & Finn, 2014].

The TrkB agonist 7,8‐dihydroxyflavone hydrate (7,8‐DHF, Sigma‐Aldrich # D5446) was given subchronically, daily for either 5 or 7 days before the start of behavioral testing, and 60 min before the start of the behavioral assay on each testing day. 7,8‐DHF was prepared fresh each morning in a vehicle of 100% dimethyl sulfoxide (DMSO, Sigma‐Aldrich #D2650) diluted with phosphate buffered saline (1% PBS, ThermoFisher #10010023) to a final concentration of 17% DMSO. Mice were treated i.p. with vehicle, 2.5, 5.0, 7.5, or 15.0 mg/kg in the BTBR experiments, and vehicle or 5 mg/kg in the *Shank3B* experiments, injected in a volume of 10 ml/kg. Doses were chosen from previous publications on behavioral actions of 7,8‐DHF in mice [Devi & Ohno, 2011; Ren et al., 2014; Castello et al., 2014; Stagni et al., 2017].

D‐cycloserine (Sigma‐Aldrich #C6880), a partial agonist at the glycine binding site on the NMDA receptor, was given acutely, 30 min before the start of the behavioral assay on each testing day. d‐Cycloserine was prepared in a vehicle of 0.9% saline. Mice were treated i.p. with vehicle, 32 mg/kg, or 320 mg/kg, injected in a volume of 10 ml/kg. Doses were chosen from previous publications on behavioral actions of d‐cycloserine in mice [Davis, Ressler, Rothbaum, & Richardson, 2006; Blundell et al., 2010; Myers & Carlezon, 2012; Benson, Burket, & Deutsch, 2013; Burket, Benson, Tang, & Deutsch, 2013; Schoch et al., 2017].

The Ampakine CX546 (Sigma‐Aldrich #C271), was given acutely, 30 min before the start of the behavioral assay on each testing day. CX546 was prepared in a vehicle of 25% β‐cyclodextrin in distilled water. Aliquots of solutions were prepared fresh prior to each test experiment and stored at 4°C. Mice were treated with vehicle or CX546 15 mg/kg, injected intraperitoneally in a volume of 10 mg/kg. The dose was chosen from previous publications on behavioral and BDNF‐mediated actions of CX546 in mice [Lynch, 2006; Lipina, Weiss, & Roder, 2007; Ogier et al., 2007; Silverman et al., 2013].

The mTOR inhibitor rapamycin (LC Laboratories, Woburn, MA, #R‐5000), was given subchronically, daily for 3 days before the start of behavioral testing, and 60 min before the start of the behavioral assay on each testing day. Rapamycin was prepared in a vehicle of 10% DMSO in PBS, stored in 6 ml aliquots at –20°C, and defrosted for 1 hr on the mornings of injections. Mice were treated with vehicle or 10 mg/kg, injected intraperitoneally in a volume of 10 ml/kg. Doses were chosen from previous publications on behavioral actions of rapamycin in mice [Ehninger et al., 2008; Tsai et al., 2012; Sato et al., 2012; Cambiaghi et al., 2013; Burket et al., 2014].

### *Statistical Analyses*

Data were analyzed with GraphPad Prism 7 (La Jolla, CA). Data requiring Three‐Way ANOVA and posthoc analyses were analyzed with Statistica Academic (Dell Software, Round Rock, TX). Results are presented as mean + standard error of the mean. Sex differences were analyzed using a main effect ANOVA. Similar scores were obtained from males and females throughout the studies, permitting combining the data from males and females, to yield larger Ns and increase statistical power. *Elevated plus‐maze*: Percent time in open arm, number of open arm entries, and number of total arm entries were analyzed using a Two‐Way ANOVA for strain differences and Tukey's multiple comparison *post hoc* test to compare strain and drug treatment groups. *Light↔dark transitions*: Time in the light chamber and number of transitions were analyzed using a Two‐Way ANOVA and Tukey's multiple comparison *post hoc. Open field*: Data from 5 min time bins were analyzed with a Three‐Way Repeated Measures ANOVA (time × genotype × treatment) and Tukey's multiple comparison *post hoc* test. Total distance within strain between treatments was analyzed by Two‐Way ANOVA and Tukey's multiple comparison *post hoc. Three‐chambered social approach*: Parameters quantified included time in each chamber during the familiarization session, number of entries into side compartments during familiarization, time in each chamber during the social approach session, time spent sniffing the novel mouse and the novel object while the subject mouse was facing and within 2 cm of the wire cups, and number of entries into side compartments during social approach, for each genotype and each treatment group. This binary, all‐or‐none test for sociability was analyzed within strain and within treatment group using One‐Way ANOVA and Tukey's multiple comparison *post hoc* test or unpaired Student's *t*‐test to compare (a) time in the chamber with the novel mouse versus in the chamber with novel object, with center time excluded, and (b) time spent sniffing the novel mouse versus time spent sniffing the novel object. In our extensive experience with the development and implementation of this assay in over 30 mutant lines and inbred strains of mice, three‐chambered social approach is not sensitive enough to compare time spent with the novel mouse across genotypes or across treatment groups. Use of a preference index can mask critical artifacts due to unusually low or high levels of exploratory activity. Therefore we consider three‐chambered social approach a yes‐or‐no measure of sociability within group. [Yang et al., 2011b; Brielmaier et al., 2014; Silverman et al., 2015]. That is, if a genotype and/or treatment group of mice spends significantly more time in the chamber with the novel mouse than in the chamber with the novel object, and significantly more time sniffing the novel mouse than the novel object, it is interpreted as displaying sociability. If time spent with the novel mouse and novel object is similar, or less time is spent with the novel mouse than with the novel object, the group fails to show sociability. More quantitative measures of social behavior require different tasks, such as male–female reciprocal social interaction, described below. Total number of entries into the two side chambers across treatments was analyzed within strain or within genotype by *t*‐test. *Self‐grooming*: Total seconds spent and number of bouts of repetitive self‐grooming were analyzed using a Two‐Way ANOVA with the factors of genotype and treatment for the *Shank3B* experiments, and Two‐Way ANOVA with the factors of strain and treatment for the BTBR experiments. In cases where the overall ANOVA was significant, Tukey's multiple comparisons *post hoc* test or unpaired Student's *t*‐test was used to compare each drug dose group to its vehicle control group. *Male–female reciprocal social interactions*: Male–female reciprocal social interactions were analyzed with a Two‐Way ANOVA for each behavioral parameter, with the factors of genotype and treatment. In cases where the overall ANOVA was significant, Tukey's multiple comparison *post hoc* test was used to compare each drug dose group to its vehicle control group. Ultrasonic vocalizations over time were analyzed with a Two‐Way ANOVA, with the factors of genotype and treatment, followed by Tukey's multiple comparison post hoc test. *Morris water maze*: Acquisition time to reach the hidden platform, distance traversed, and swim speed were analyzed using Three‐Way ANOVA and Tukey's multiple comparisons *post hoc*. Probe trial data were analyzed using a One‐Way ANOVA and Tukey's multiple comparisons *post hoc* within each genotype, strain, and treatment group, for the parameters of time spent in each quadrant and number of crossings over the previous training platform location and corresponding platform locations in the other three quadrants. *Sex comparisons*: Three‐Way ANOVAs, in which sex of the subject mice was one factor, were separately conducted to evaluate sex as a biological variable. No consistent phenotypic differences between males and females were detected in any of the assays with these genotypes or inbred strains.

## Results

### *GABA‐A Agonist Reduced Repetitive Self‐Grooming in BTBR Mice*

**Figure 1:**
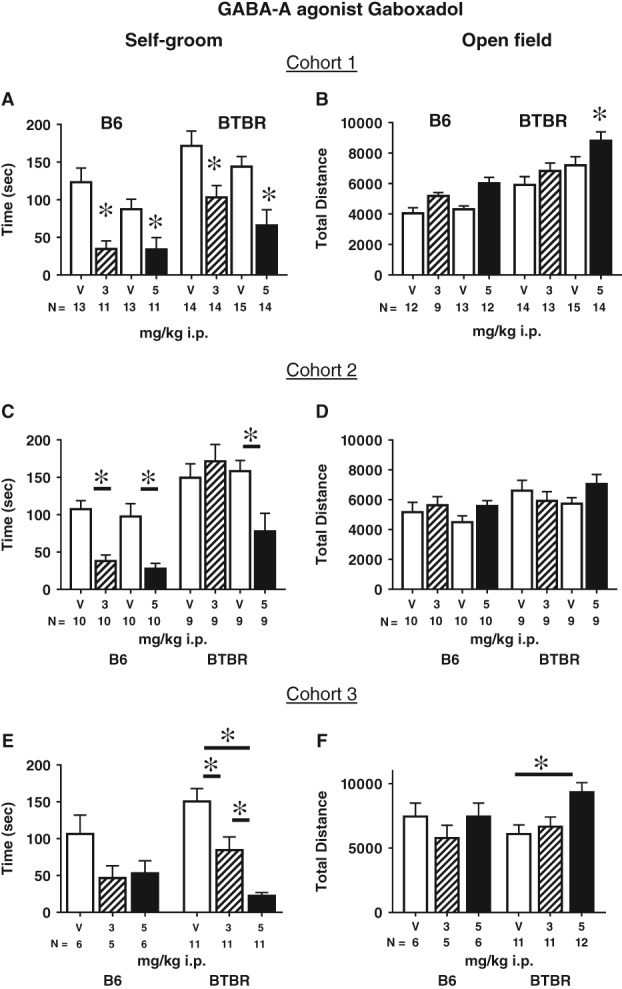
Gaboxadol, an extrasynaptic GABA‐A receptor agonist, significantly reduced the high levels of spontaneous repetitive self‐grooming in the BTBR T+ Itpr3tf/J (BTBR) mouse model of idiopathic autism, at doses that were not sedating. Gaboxadol reversal of this repetitive behavior in BTBR was replicated in three independent cohorts. Two doses, 3 mg/kg and 5 mg/kg, or saline vehicle were administered intraperitoneally, acutely, 30 min before the start of each behavioral test, to the control strain C57BL/6 J (B6). Grooming was also reduced in B6. *Cohort 1*: BTBR mice displayed more self‐grooming than B6 controls, as previously reported (Two‐Way ANOVA, genotype: F_1,97_ = 18.55, *P* < 0.001). Repetitive self‐grooming was reduced by gaboxadol in both BTBR and B6 mice (F_7, 97_ = 8.607, *P* < 0.001). (A) Total time spent in self‐grooming: B6, Tukey's multiple comparisons post‐hoc *P < 0.05 vehicle versus 3 mg/kg; *P < 0.05 vehicle versus 5 mg/kg; BTBR, *P < 0.05 vehicle versus 3 mg/kg *P < 0.05, **P < 0.01 vehicle versus 5 mg/kg. (B) Open field exploratory locomotion was increased by the higher dose of gaboxadol (F_7.97_ = 11.2, *P* < 0.001), B6, NS; BTBR, *P < 0.01 vehicle versus 5 mg/kg. *Cohort 2*: Self‐grooming was higher overall in BTBR than B6 (F_1,68_ = 39.5, *P* < 0.001). Self‐grooming was reduced by gaboxadol (F_7, 68_ = 11.31, *P* < 0.0001). (C) Time spent grooming: B6, *P < 0.05 vehicle versus 3 mg/kg, *P < 0.05 vehicle versus 5 mg/kg; BTBR, NS vehicle versus 3 mg/kg, *P < 0.05 vehicle versus 5 mg/kg. (D) Open field exploratory locomotion was not significantly affected by gaboxadol treatment in either strain at either dose (F_7,67_ = 2.028, *P* = 0.0641, NS). *Cohort 3*: Self‐grooming was reduced by gaboxadol in BTBR mice (F_5, 44_ = 8.769, *P* < 0.001), at doses that increased open field locomotion only at the higher dose (F_5, 45_ = 2.683, *P* = 0.0332). (E) Time spent grooming: B6, NS; BTBR, *P < 0.05 vehicle versus 3 mg/kg, *P < 0.05 vehicle versus 5 mg/kg. (F) Open field activity: B6, NS; BTBR, open field activity was increased by the higher dose of gaboxadol: NS vehicle versus 3 mg/kg, *P < 0.05 vehicle versus 5 mg/kg. In all figures, data are expressed as mean + standard error of the mean, with the number of mice per group displayed on the x‐axis of each graph.

Based on reports of low GABAergic inhibitory transmission, reduced BDNF, ERK1, and elevated mTOR in the BTBR strain as compared to a standard C57BL/6 J (B6) control strain [Gogolla et al., 2009; Stephenson et al., 2011; Burket et al., 2014; Han, Tai, Jones, Scheuer, & Catterall, 2014; Seese et al., 2014; Daimon et al., 2015; Steinmetz, Stern, Kohtz, Descalzi, & Alberini, 2018], we hypothesized that increasing GABAergic neurotransmission might improve social behaviors and reduce repetitive behaviors in BTBR. We first focused on a pharmacological target that could increase inhibitory neurotransmission in BTBR. Gaboxadol (formerly THIP) is an agonist at extrasynaptic GABA‐A receptors containing α4βδ subunits, that exerts tonic inhibition [Waszczak, Hruska, & Walters, 1980; Brown, Kerby, Bonnert, Whiting, & Wafford, 2002; Iversen, 2004; Farrant & Nusser, 2005; Meera, Wallner, & Otis, 2011; Carver & Reddy, 2013; Olson, 2018]. OV101, a gaboxadol formulation, is in clinical trials for Angelman syndrome (https://clinicaltrials.gov/ct2/show/NCT02996305). BTBR and B6 mice were treated acutely with saline vehicle or gaboxadol, 3 mg/kg or 5 mg/kg, administered intraperitoneally 30‐min before the start of each autism‐relevant behavioral assay: Three‐chambered social approach, male–female reciprocal social interactions, and repetitive self‐grooming in an empty cage. B6 was employed as the standard control strain, based on its reproducibly high levels of sociability and comparatively low levels of repetitive behaviors [Bolivar et al., 2007; Moy et al., 2007; Yang et al., 2007; McFarlane et al., 2008; Pobbe et al., 2010; Pearson et al., 2011, 2012; Gould et al., 2012]. Gaboxadol significantly reduced the high level of self‐grooming that characterizes BTBR (Fig. 1). The ability of gaboxadol to reduce repetitive behavior was significant in three separate full cohorts of BTBR mice, at the 5 mg/kg dose in all three cohorts (Fig. 1A,C,E), and at the 3 mg/kg dose in two cohorts (Fig. 1A,E). B6 mice similarly displayed lower levels of self‐grooming after gaboxadol treatment (Fig. 1A,C,E). To address the possibility that the reduction in self‐grooming was an artifact of sedation induced by the GABA agonist, the same doses were administered to the same mice 1 week later on a separate 30 min open field exploratory locomotion assay, as a control for general exploratory activity. Gaboxadol did not reduce general activity levels at either dose in either strain (Fig. 1B,D,F), and in fact elevated activity at the higher dose, ruling out the potential confound of sedation. Gaboxadol at these doses did not produce consistently significant effects on the two social assays (Supporting Information Figs. S1 and S2). It is important to note that, paradoxically, BTBR mice did not display their usual social deficits in these experiments, for unknown reasons that will require further investigation. Gaboxadol at these doses in these lines of mice did not produce its predicted anxiolytic effect on elevated plus‐maze (Supporting Information Figs. S3 and S4) or light↔dark transitions (Supporting Information Fig. S5). Strain differences were detected on parameters that indicate less anxiety‐like behaviors in BTBR treated with vehicle as compared to B6 treated with vehicle. Robust reductions in a repetitive behavior by gaboxadol, replicated in three independently bred cohorts of BTBR mice, with no indication of sedation, support the possibility that a GABAergic agonist such as gaboxadol could offer a pharmacological target for reducing repetitive behaviors in autism.

### *TrkB Agonist Improved Sociability in BTBR Mice*

**Figure 2:**
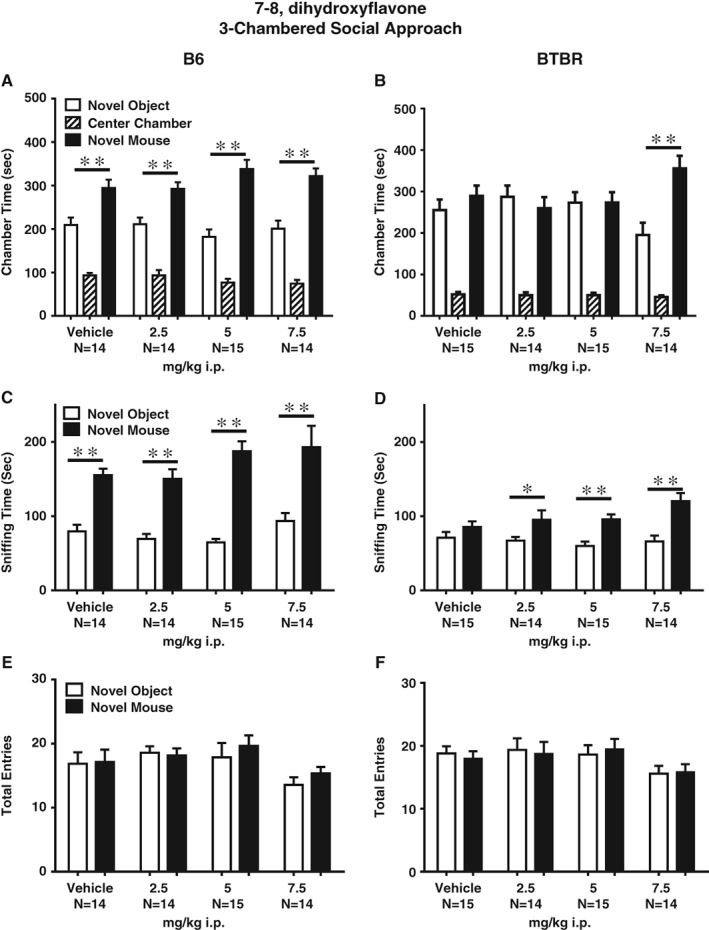
The TrkB receptor agonist 7,8‐dihydroxyflavone (7,8‐DHF), reversed the social deficit in BTBR mice on the three‐chambered social approach assay, with no deleterious effects in B6 control mice. 7,8‐DHF in a vehicle of phosphate buffered saline and 17% DMSO was administered intraperitoneally for 7 days, and 60 min prior to the behavioral testing session. (A) B6 mice displayed normal sociability, spending more time in the side chamber with the novel mouse than in the side chamber with the novel object (F_7, 104_ = 44.76, *P* < 0.0001), in the groups treated with vehicle, 2.5 mg/kg, 5.0 mg/kg, and 7.5 mg/kg 7,8‐DHF (**P < 0.01 chamber time for all vehicle versus treatment comparisons using Tukey's multiple comparison post‐hoc analysis). (B) BTBR mice treated with vehicle failed to display normal sociability, spending equal time in the side chamber with the novel mouse and in the side chamber with the novel object, as previously reported. BTBR treated with 2.5 and 5.0 mg/kg 7,8‐DHF similarly failed to display sociability on the chamber time parameter. BTBR mice treated with 7.5 mg/kg 7,8‐DHF displayed normal sociability, spending more time in the side chamber with the novel mouse than in the side chamber with the novel object (F_7, 108_ = 2.675, *P* = 0.0136, **P < 0.01). (C) On the more sensitive social parameter of time spent sniffing the novel mouse versus time spent sniffing the novel object, B6 mice displayed normal sociability in all treatment groups (**P < 0.01 for vehicle, 2.5 mg/kg, 5.0 mg/kg, 7.5 mg/kg). (D) BTBR mice treated with vehicle failed to display normal sociability on sniff time, as previously reported. Treatment with 7,8‐DHF restored sociability on the sniff time parameter at doses of 2.5 mg/kg (*P < 0.05), 5.0 mg/kg (**P < 0.01), and 7.5 mg/kg (**P < 0.01). (E,F) Number of entries into the side chambers, a control measure of general exploratory activity in the three‐chambered apparatus, confirmed no effect of 7,8‐DHF on exploratory locomotion in either strain in any treatment group. No sex differences were detected.

We next considered the low BDNF levels in BTBR mice, reasoning that activation of the tropomyosin‐related kinase‐B (TrkB) receptor, which mediates the actions of BDNF [Massa et al., 2010], could ameliorate behavioral abnormalities resulting from impaired synaptic signaling. BDNF regulates neuronal growth and differentiation, synaptic plasticity, learning and memory, and is implicated in mood disorders and responses to aversive social experiences [Berton et al., 2006; Björkholm & Monteggia, 2016; Mitre, Mariga, & Chao, 2017; Rosas‐Vidal et al., 2018]. 7,8‐dihydroxyflavanone (7,8‐DHF), a small molecule flavonoid that mimics the actions of BDNF by binding to and activating its TrkB receptor, was administered intraperitonally to BTBR and B6 control mice, daily beginning a week before the start of behavioral testing, and 60 min before the start of each testing session. Vehicle‐treated B6 mice displayed normal sociability (Fig. 2A) while BTBR mice displayed the expected absence of sociability (Fig. 2B) on three‐chambered social approach. 7,8‐DHF restored sociability in BTBR at the 7.5 mg/kg dose on the chamber time parameter (Fig. 2B), and restored sociability at doses of 2.5, 5.0, and 7.5 mg/kg on the social sniffing parameter (Fig. 2D). In contrast, 7,8‐DHF did not affect the normal sociability in B6 mice (Fig. 2A,C), indicating a selective effect of 7,8‐DHF in BTBR on only one social behavior assay. The treatment regimen had no effect on number of entries into the side chambers, an internal measure of general exploratory activity, and had no effect on general exploratory locomotion in an open field (Supporting Information Fig. S6), indicating no deleterious side effects of this treatment regimen on motor functions. In contrast, on male interactions with an estrous female, a second test for sociability, 7,8‐DHF at these doses did not improve the parameters of social interaction in which BTBR was lower than B6 (Supporting Information Fig. S7). In addition,7,8‐DHF did not reduce self‐grooming in BTBR (Supporting Information Fig. S8). These findings indicate a selective rather than a broad effect of a TrkB agonist on one social assay in BTBR mice.

### *Rapamycin Did Not Significantly Reduce Repetitive Behavior in BTBR*

Given that elevated mTOR signaling has been identified in the BTBR inbred strain, the third pharmacological target evaluated in BTBR was rapamycin, a well‐established mTOR inhibitor. mTOR is a key element in signaling pathways that regulate cell growth, proliferation, autophagy, and dendritic spine morphology, and has been implicated in neurodevelopmental disorders including autism, intellectual disabilities, and epilepsy [Crino, 2011; Bateup et al., 2013; Phillips & Pozzo‐Miller, 2015; Henry, Hockeimer, Chen, Mysore, & Sutton, 2017]. Two independent cohorts of BTBR and B6 controls were treated subchronically with vehicle or rapamycin 10 mg/kg i.p. A trend for reduced repetitive self‐grooming was seen in BTBR Cohort 1, but not in BTBR Cohort 2 (Supporting Information Fig. S9). Rapamycin had no effect on social behaviors in B6 or BTBR (Supporting Information Figs. S10 and S11), and no consistent effect on open field activity, in either cohort of B6 and BTBR (Supporting Information Fig. S12). From these studies in the BTBR mouse model of idiopathic autism, it appears that mTOR inhibition is not a promising target for intervention.

### *D‐Cycloserine, CX546 and 7,8‐DHF in Shank3B Mice*

Evidence for reduced excitatory neurotransmission, long‐term potentiation, NMDA/AMPA ratio, and dendritic spine abnormalities in *Shank3B* mice formed the basis of our hypothesis that pharmacologically increasing glutamatergic neurotransmission could improve social behaviors and/or reduce repetitive behaviors in *Shank3B*. To investigate the possibility that enhancing glutamatergic synaptic transmission and increasing glutamatergic excitation could reverse autism‐relevant phenotypes in *Shank3B* mice [Lovinger, 2017; Wang et al., 2017], three classes of pharmacological compounds were evaluated in *Shank3B* and their wildtype littermate controls: (a) a partial agonist at the glycine modulatory site on the NMDA receptor, d‐cycloserine, that has been evaluated preclinically and in clinical trials for several neuropsychiatric disorders [Baxter et al., 1994; Myers & Carlezon, 2012; Schade & Paulus, 2016]; (b) an Ampakine positive allosteric modulator of the glutamatergic AMPA receptor, CX546, one of a class of compounds found to improve cognition in rodents and tested in clinical trials for schizophrenia and Fragile X syndrome [Goff et al., 2001; Berry‐Kravis et al., 2006; Arai & Kessler, 2007; Lynch, Palmer, & Gall, 2011]; and (c) the TrkB agonist 7,8‐DHF, as described above. Two behavioral assays in which *Shank3B* mice were previously reported to display robust and replicated deficits were employed as outcome measures, male–female reciprocal social interactions and repetitive self‐grooming, along with open field exploratory locomotion as a control for the potential confounds of sedation and hyperactivity after drug treatments.

**Figure 3:**
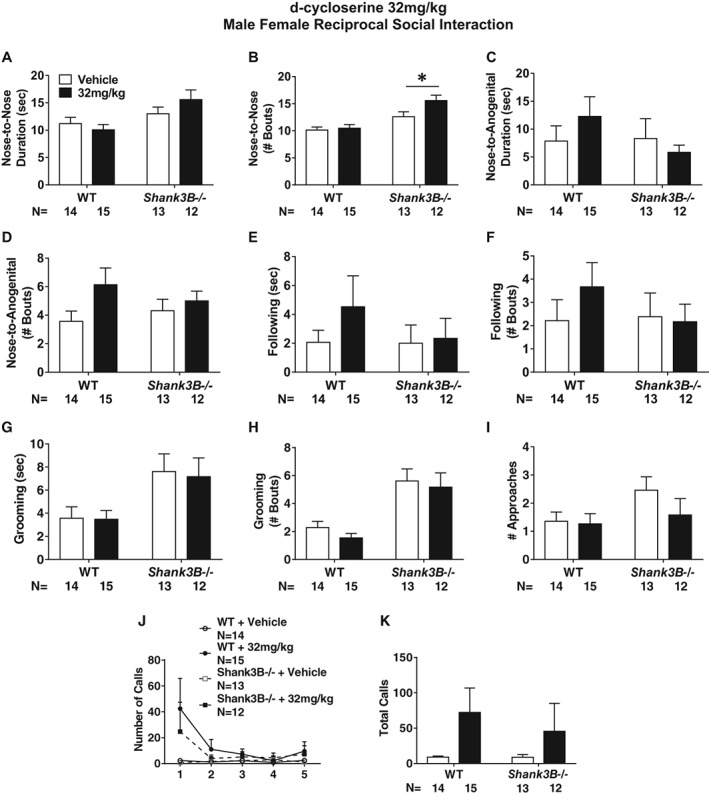
D‐cycloserine, a partial agonist at the glycine binding site on the glutamatergic NMDA receptor, given at a dose of 32 mg/kg, increased one parameter of male–female reciprocal social interaction in *Shank3B* null mutant mice, bouts of nose‐to‐nose sniffing, and had no significant effects in wildtype (WT) littermate controls. (A) Time spent engaged in nose‐to‐nose sniffing was significant for genotype (Two‐Way ANOVA F_1,50_ = 8.335, *P* < 0.01), but no effect of drug treatment was detected (F_1,50_ = 0.322, NS). (B) Number of bouts of nose‐to‐nose sniffing was significant for genotype (F_1,50_ = 23.69, *P* < 0.001) and significant for treatment (F_1,50_ = 4.457, *P < 0.05). At the 32 mg/kg dose, d‐cycloserine had no effect in WT or *Shank3B* mice on (C) time spent in nose‐to‐anogenital sniffing, (D) bouts of nose‐to‐anogenital sniffing, (E) time spent following, (F) number of following bouts. (G) Self‐grooming time was higher in *Shank3B* than WT (F_1,50_ = 26.1, *P* < 0.001), but unaffected by d‐cycloserine treatment (F_1,50_ = 0.7766, *P* = 0.382). (H) Self‐grooming bout numbers were higher in *Shank3B* than WT (F_1,50_ = 26.1, *P* < 0.001), but unaffected by d‐cycloserine treatment (F_1,50_ = 0.777, NS). (I) Number of approaches was unaffected by treatment (F_1,50_ = 1.245, *P* = 0.2698, NS). (J) Ultrasonic vocalizations showed no treatment effect when measured in 1 min time bins (F_3, 50_ = 1.468, NS). (K) A nonsignificant trend toward more vocalizations after treatment overall was detected when ultrasonic vocalizations were summed across the 5‐min test session (F_1, 50_ = 3.64, *P* = 0.0622).

D‐cycloserine at an acute dose of 32 mg/kg improved social scores on number of bouts of nose‐to‐nose sniffing, but not on other parameters measured during male *Shank3B* interactions with an estrous B6 female (Fig. 3). A higher dose of d‐cycloserine, 320 mg/kg, previously reported to improve social behaviors in mouse models of autism [Burket et al., 2013; Benson et al., 2013], significantly increased three out of four parameters of male *Shank3B* social interactions with an estrous B6 female (Supporting Information Fig. S13). It is important to note that male–female interaction scores were not lower in *Shank3B* than in WT in these cohorts. Further, open field activity was dramatically elevated in both WT and *Shank3B* at the 320 mg/kg dose (Supporting Information Fig. S14). Hyperlocomotion appeared to be responsible for the increased contact with the female, as the subject males were observed to encounter the novel female more frequently while moving rapidly around the testing arena. Hyperlocomotion at the 320 mg/kg dose may be consistent with findings that high doses of d‐cycloserine have antagonist actions at NMDA receptors [Lanthorn, 1994]. The low dose of d‐cycloserine did not affect self‐grooming, whereas 320 mg/kg d‐cycloserine reduced the high level of self‐grooming in *Shank3B* mice (Supporting Information Fig. S15). The reduction was likely a consequence of a competing behavior, that is, competition with high levels of exploratory behavior.

**Figure 4:**
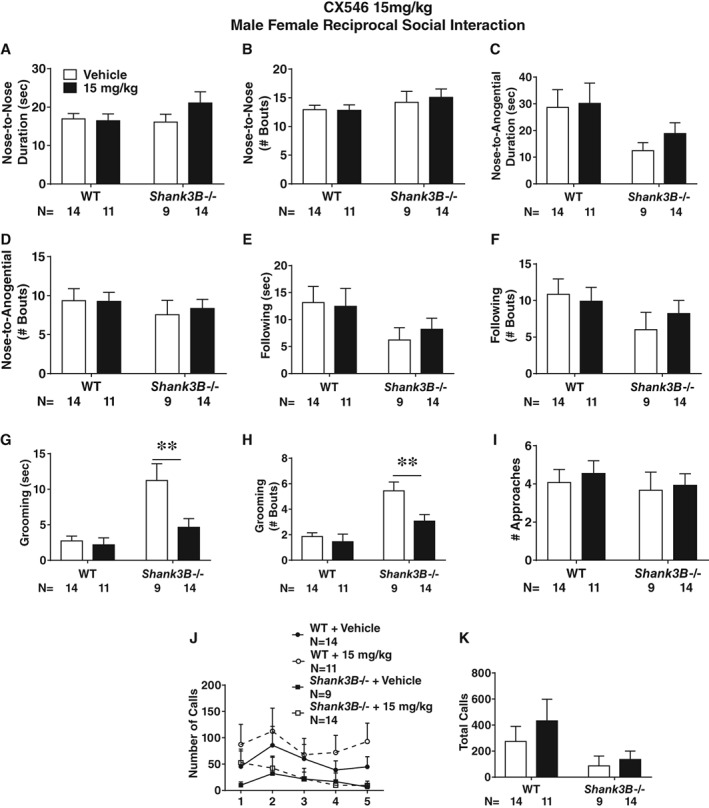
The ampakine CX546, a positive allosteric modulator of the glutamatergic AMPA receptor, administered intraperitoneally to male WT and *Shank3B* mice at a dose of 15 mg/kg, 30 min before the start of behavioral testing, had no effect on male–female reciprocal social interactions. CX546 significantly reduced self‐grooming during the social test session in *Cohort 1 Shank3B* null mutant mice. (A) Nose‐to nose sniffing by male *Shank3B* did not differ significantly from male WT (Two‐Way ANOVA, genotype F_1,44_ = 0.729, NS). No effect of drug treatment was detected (F_1,44_ = 1.016, NS). (B) Number of bouts of nose‐to‐nose sniffing showed no effect of genotype (F_1,44_ = 1.838, NS), or treatment (F_1,44_ = 0.079, NS). (C) *Shank3B* spent less time than WT in nose‐to‐nose sniffing (F_1,44_ = 5.373, *P* < 0.05), which was not significantly increased by drug treatment (F_1,44_ = 0.449, NS). (D) Number of bouts of nose‐to‐nose sniffing did not differ between genotypes (F_1,44_ = 0.881, NS) or treatments (F_1,44_ = 0.061, NS). (E) *Shank3B* showed a trend toward less time following the female than WT (F_1,44_ = 3.96, *P* = 0.053), with a trend toward more following after drug treatment (F_1,44_ = 0.054, NS). (F) Number of bouts of following did not differ for genotype (F_1,44_ = 2.495, NS) or treatment (F_1,44_ = 0.093, NS). (G) Time spent grooming during the male–female social interaction test was higher in *Shank3B* than WT (F_1,44_ = 17.7, *P* < 0.001). The high level of self‐grooming in *Shank3B* during the male–female interaction session was significantly reduced by CX546 treatment (F_1,44_ = 7.44, *P* < 0.01; Tukey's post hoc comparison significant for *Shank3B* vehicle versus *Shank3B* CX546, **P < 0.01). (H) Number of bouts of self‐grooming was similarly higher in *Shank3B* than WT (F_1,44_ = 25.5, *P* < 0.001). CX546 treatment significantly reduced number of grooming bouts in *Shank3B* (F_1,44_ = 7.25, **P < 0.01, *Shank3B* vehicle versus *Shank3B* CX546, *P < 0.05). (I) Total number of approaches did not differ between genotypes (F_1,44_ = 0.496, NS) or treatments (F_1,44_ = 0.257, NS). Ultrasonic vocalizations showed no treatment effect when measured in (J) 1 min time bins (F_1,44_ = 0.257, NS), or (K) summed across the 5‐min test session (F_1,44_ = 0.824, NS). Supporting Information Figures S16, S17, S18 indicate lack of replication of the effects of CX546 in *Shank3B Cohort 2* on grooming during male–female interactions, and in a separate repetitive self‐grooming assay, along with a caveat concerning locomotor activity.

CX546 at an acute dose of 15 mg/kg given 30 min before the start of each behavioral testing session had no effect on parameters of male–female social interaction, but significantly reduced self‐grooming in one cohort of *Shank3B* during the social interaction session (Fig. 4). However, a second cohort of *Shank3B* did not show reductions in self‐grooming during the social test with the same CX546 treatment (Supporting Information Fig. S16), and the high levels of self‐grooming in *Shank3B* during the standard empty cage test for repetitive behaviors were not reduced by CX546 treatment in either cohort (Supporting Information Fig. S17). It is possible that the older age of Cohort 1 mice used in this study, 6–7 months, was more sensitive to CX546 than the 2–3 month olds in Cohort 2. *Shank3B* displayed low scores on open field locomotion as previously reported [Dhamne et al., 2017], however, no drug effects were detected on open field exploratory activity (Supporting Information Fig. S18).

**Figure 5:**
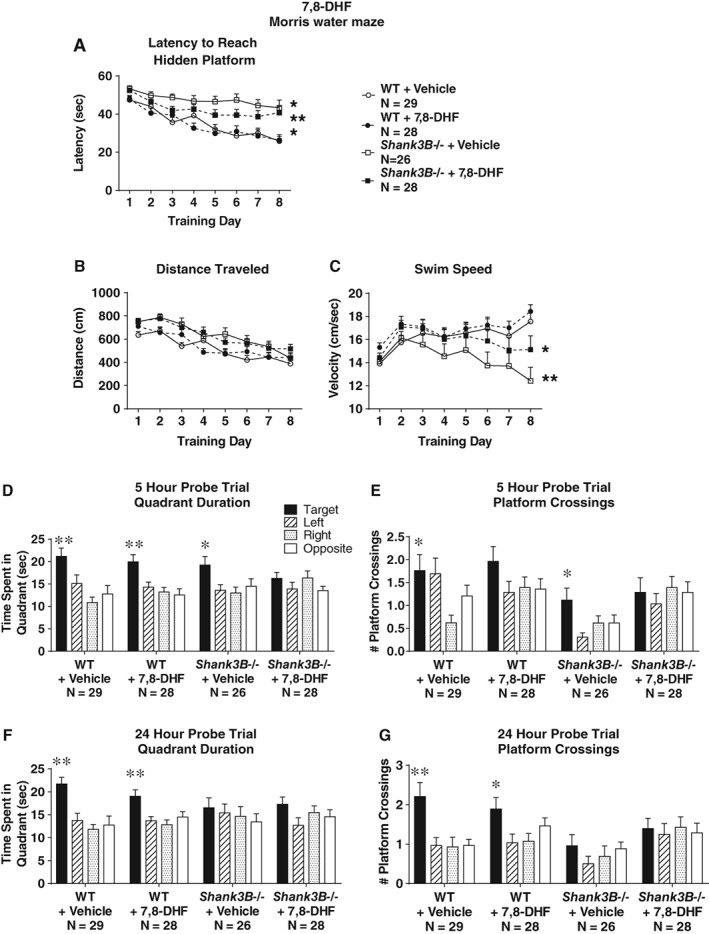
The TrkB receptor agonist 7,8‐dihydroxyflavone (7,8‐DHF), improved components of the deficits in Morris water maze spatial learning in *Shank3B* null mutant mice. 7,8‐DHF was administered intraperitoneally at a dose of 5 mg/kg, daily for 5 days before the start of water maze training and 60 min before the start of each training session. (A) Latency to reach the hidden platform was significantly slower for *Shank3B* than for WT littermates (Three‐Way ANOVA, genotype: F_3,440_ = 31.67, **P < 0.001). 7,8‐DHF improved acquisition in *Shank3B* as compared to vehicle‐treated *Shank3B* (treatment: F_3,416_ = 12.75, **P < 0.001), showing a small but significant improvement in the learning curve, although neither *Shank3B* group reached a final latency below 40 sec. No significant effect of 7,8‐DHF was detected in WT (treatment: F_3,440_ = 0.806, NS). (B) Distance traveled to reach the hidden platform did not differ between genotypes (F_1,440_ = 1.596, NS) and was not affected by 7,8‐DHF treatment in either WT (F_1,440_ = 1.596, NS) or *Shank3B* (F_1,416_ = 0.0159, NS). (C) Swim speed was lower in *Shank3B* as compared to WT (F_3,440_ = 8.13, **P < 0.001), consistent with lower open field activity in *Shank3B* mice as shown in Supplementary Figure 21. 7,8‐DHF increased swim speed in *Shank3B* (F_7,440_ = 4.378, *P < 0.05). Faster swimming after 7,8‐DHF treatment may have contributed to the apparent improvement in the learning curve for *Shank3B* mice treated with 7,8‐DHF. (D) Probe trial performance 5 hr after the last training trial, using Dunnett's multiple comparison test, showed significantly more time spent in the pool quadrant which previously contained the hidden platform as compared to the other three quadrants for WT + vehicle (F_3,112_ = 1.171, **P < 0.01), WT + 7,8‐DHF (F_3,108_ = 1.509, **P < 0.01), and *Shank3B* + vehicle (F_3,100_ = 1.508, *P < 0.05), but not for *Shank3B* + 7,8‐DHF (F_3,108_ = 0.788, NS). (E) Probe trial performance 5 hr after the last training trial showed significantly more crossings over the previous location of the hidden platform as compared to the corresponding locations in the other three quadrants for WT + vehicle (F_3,112_ = 2.539, *P < 0.05), but not for WT + 7,8‐DHF (F_3,108_ = 1.756, *P*= 0.228, NS), and for *Shank3B* + vehicle (F_3,100_ = 3.984, *P < 0.05), but not for *Shank3B* + 7,8‐DHF (F_3,108_ = 0.926, NS). (F) Probe trial performance 24 hr after the last training trial showed significantly more time spent in the pool quadrant which previously contained the hidden platform as compared to the other three quadrants for WT + vehicle (F_3,112_ = 5.107, **P < 0.001), WT + 7,8‐DHF (F_3,108_ = 8.032, **P < 0.01), but not for *Shank3B* + vehicle (F_3,100_ = 0.720, NS), or *Shank3B* + 7,8‐DHF (F_3,108_ = 0.232, NS). (G) Probe trial performance 24 hr after the last training trial showed significantly more crossings over the previous location of the hidden platform as compared to the corresponding locations in the other three quadrants for WT + vehicle (F_3,112_ = 4.200, **P < 0.001), WT + 7,8‐DHF (F_3,108_ = 1.557, *P < 0.05), but not for *Shank3B* + vehicle (F_3,100_ = 0.740, NS), or *Shank3B* + 7,8‐DHF (F_3,108_ = 0.284, NS). Similar profiles were seen in males and females.

7,8‐DHF, 5 mg/kg given intraperitonally daily beginning 5 days before the start of behavioral testing, and 60 min before the start of each testing session, did not increase the low scores of *Shank3B* on parameters of male–female social interactions (Supporting Information Fig. S19). Self‐grooming in an empty cage was not significantly affected by 7,8‐DHF treatment, although a trend for a reduction was seen in males only (Supporting Information Fig. S20). Open field exploration was unaffected by 7,8‐DHF, confirming absence of sedation or hyperactivity with this treatment regimen (Supporting Information Fig. S21). As deficits in Morris water maze acquisition were previously reported in *Shank3B* mice [Dhamne et al., 2017], consistent with their reduced hippocampal long‐term potentiation, and as TrkB agonists have been reported to improve learning and memory [Devi & Ohno, 2011; Bollen et al., 2013; Castello et al., 2014], 7,8‐DHF was tested on water maze learning in WT and *Shank3B*. Significant improvement in acquisition of the location of the hidden platform was detected in *Shank3B* treated with 7,8‐DHF, with no deleterious effects on performance in WT (Fig. 5A). It is important to note that low swim speeds seen in *Shank3B* were increased by 7,8‐DHF (Fig. 5C), a finding which offers an alternative explanation, that is, faster swimming after drug treatment, for the apparent improved learning in the water maze spatial task. However, total distance traveled was unaffected by drug treatment (Fig. 5B). Probe trial measures of memory were not improved (Fig. 5D–G).

## Discussion

Neurodevelopmental disorders caused by known biological abnormalities are potentially amenable to targeted medical therapeutics. Clinical trials of pharmacological interventions for autism are currently at an early stage, with questions to be resolved about outcome measures, experimental design, age of treatment onset, duration of treatment, and patient stratification, for example, by language skills, IQ, or biomarkers [Jacquemont et al., 2018]. Careful preclinical studies, employing robust phenotypes in animal models with high construct validity, can build confidence in pharmacological targets designed to reverse behavioral deficits based on biological abnormalities identified in the model system [Spooren et al., 2012; Kazdoba et al., 2016a; Chadman, 2017].

We evaluated five classes of drugs in two distinct mouse models of autism, using behavioral assays selected for high relevance to the core diagnostic symptoms of autism. Only robust phenotypes that were reported previously and replicated in the current studies were employed, to ensure a sufficiently strong and invariable signal to detect bone fide reversal by drug treatments. The primary discovery was reductions in excessive repetitive behavior by the GABA‐A agonist gaboxadol, replicated in three independent cohorts of BTBR mice. This strong finding suggests that GABA agonists targeting impairments in inhibition, perhaps through extrasynaptic receptors that mediate tonic inhibition, may offer a potential therapeutic target for people with autism in whom extreme repetitive behaviors are problematic. Reductions in repetitive self‐grooming by gaboxadol, replicated in three separate cohorts of BTBR mice, offer proof‐of‐principle for the advantages of rigorous preclinical experimental design in generating reproducible findings.

Additional intriguing findings were obtained with a TrkB agonist, postulated to improve synaptic transmission through a neurotrophic pathway activated by BDNF. Evidence for reversal of some aspects of low sociability in BTBR mice, and improvement in water maze learning in *Shank3B* mice, raises the possibility that pharmacological interventions designed to more selectively activate TrkB receptors and pathways downstream to the actions of BDNF [Kron et al., 2014; Merkouris et al., 2018], or to enhance synapse development and plasticity more generally, may be beneficial. Partial or fully negative findings obtained with three other compounds, rapamycin, CX546, and d‐cycloserine, which were proposed to normalize dysregulated signaling cascades, emphasize the usefulness of comparing pharmacological targets within the same animal models using the same assays. A matrix of results from multidisciplinary assays by many labs, across many animal models and for many classes of pharmacological targets, could provide informative comparisons to guide the interpretations of preclinical discoveries. Especially for neurodevelopmental disorders where no medical treatment exists, ruling out ineffective pharmacological targets is essential to the process of discovering positive hits, in the search for effective pharmacological therapeutics.

Consideration of caveats serves to sharpen the interpretation of behavioral pharmacology studies. While doses and treatment regimens employed in the present experiments were chosen from the literature, further analyses with dose–response curves, and comparisons of acute, semi‐chronic, and chronic administration, will be necessary to definitively confirm findings. Potential artifacts, such as the reduced swim speed in *Shank3B* mice that was reversed by treatment with the TrkB agonist, and the striking hyperactivity induced by the high dose of d‐cycloserine, may limit an interpretation of these drug effects as specific to social and cognitive improvements. Lack of drug effects on the low vocalization scores in *Shank3B* mice raises questions about the usefulness of this behavior for detecting pharmacological reversals. Normal sociability in some cohorts of BTBR was unexpected and unexplained, requiring further investigations, and reflecting the need to confirm phenotypes before drug testing. Significant treatment effects in one out of two cohorts, such as CX546 reductions in repetitive self‐grooming in cohort 1 but not in cohort 2, may best be interpreted as a minor or suggestive improvement that requires further replication. Reversal of only some parameters of social deficits in a mouse model of autism may indicate a promising lead that requires a second or third corroborative behavioral assay for full confirmation. Differences observed between cohorts, and across parameters within a behavioral assay, are not uncommon in preclinical studies. Replication of an initial finding is therefore a necessary second step, to determine the strength of a pharmacological effect. Focusing on the most robust, well‐replicated phenotypes may help to avoid disappointments along the long translational road from exploratory mouse studies to successful human clinical trials.

The present studies offer an initial behavioral investigation of pharmacological targets in two prominent mouse models of autism in which autism‐relevant behavioral phenotypes are well established. *Shank3B* was selected to model the comparatively high prevalence of *SHANK3* mutations in autism and other neurodevelopmental disorders, and the non‐monogenic BTBR strain was selected to model a subset of the cases of autism that are idiopathic, that is, heterogeneous with no identified genetic and/or environmental etiology. Extending our systematic approach to a broader range of genetic and environmental animal models of autism, as well as a broader range of hypothesis‐based pharmacological classes, may enhance future preclinical discovery. For example, although the present mouse models showed mostly similar results in males and females, testing other animal models with reported sex differences may add to the power of findings, given the higher prevalence of autism in boys versus girls. Importantly, the present experiments focused on behavioral outcome measures only. Mechanistic studies of biological abnormalities in mouse models, including measures of synaptic morphology, electrophysiology, biochemistry, and neuroanatomy, along with in vivo physiology in awake mice and humans, will be necessary to confirm and understand the mechanisms underlying pharmacological improvements in autism‐relevant behaviors.

Several lines of evidence indicate that targeting various GABA receptor subtypes and allosteric modulatory sites may ameliorate autism‐relevant phenotypes in mouse models of autism. These include our previous reports that a GABA‐B agonist, r‐baclofen, improved social behaviors and reduced repetitive behaviors in BTBR and C58/J mice [Silverman et al., 2015], and improved social and cognitive deficits in 16p11.2 deletion mice [Stoppel et al., 2018], and several reports that low doses of benzodiazepines ameliorated autism‐relevant behavioral symptoms in BTBR [Pobbe et al., 2011; Defensor et al., 2011; Han et al., 2014; Yoshimura et al., 2017]. Therapeutics that increase GABAergic neurotransmission offer potential targets for treating symptoms of autism [Lemonnier et al., 2012; Erickson et al., 2014; Mohler, 2015; Veenstra‐VanderWeele et al., 2017], particularly in individuals with debilitating repetitive behaviors, seizures, unusual profiles of EEG physiology, mutations in GABA receptor genes, mutations which reduce the development or functions of GABAergic interneurons, and other indicators of impaired inhibitory neurotransmission.

## Author contributions

MAR conducted the BTBR gaboxadol and rapamycin experiments, JMP conducted all *Shank3B* experiments, TMK conducted the BTBR TrkB experiments. MAR, JMP, and TMK contributed to the experimental design, data analyses and graphical presentation of the data, and wrote sections of the manuscript. MNS conducted statistical analyses of the data and standardized figures. JNC conceived of the project, contributed to the experimental design, established protocols, supervised the conduct of experiments, and wrote the manuscript.

## Competing interests

All authors declare no competing interests. No industry involvement or funding supported these studies. All compounds were purchased from commercial sources.

## Supporting information


**Figure S1**. The GABA‐A agonist gaboxadol had no significant effects on three‐chambered social approach in BTBR mice.
**Figure S2**. The GABA‐A agonist gaboxadol increased only one parameter of male–female reciprocal social interactions in BTBR.
**Figure S3**. Gaboxadol had no significant effects on elevated plus‐maze, Cohort 1.
**Figure S4**. Gaboxadol had no significant effects on elevated plus‐maze, Cohort 2.
**Figure S5**. Gaboxadol had no significant effects on light↔dark transitions.
**Figure S6**. The TrkB receptor agonist 7,8‐dihydroxyflavone (7,8‐DHF), had no effect on open field exploratory locomotion in BTBR.
**Figure S7**. 7,8‐DHF did not reverse the social deficit in BTBR mice in male–female reciprocal social interactions.
**Figure S8**. 7,8‐DHF did not reduce the high levels of repetitive self‐grooming in BTBR.
**Figure S9**. The mTOR inhibitor rapamycin did not reduce the high levels of repetitive self‐grooming in BTBR.
**Figure S10**. Rapamycin did not affect three‐chambered social approach.
**Figure S11**. Gaboxadol had no effect on parameters of male–female reciprocal social interactions.
**Figure S12**. Rapamycin had no consistent effects on open field exploratory locomotor behavior.
**Figure S13**. D‐cycloserine, an agonist of the glycine‐B site on the NMDA receptor, 320 mg/kg, increased parameters of male–female reciprocal social interaction in *Shank3B* null mutant mice.
**Figure S14**. D‐cycloserine 320 mg/kg induced hyperlocomotion.
**Figure S15**. D‐cycloserine reduced the high levels of self‐grooming in *Shank3B* mice only at the high dose which induced hyperlocomotion.
**Figure S16**. Ampakine CX546 had no effect on parameters of social and self‐grooming behaviors during male–female reciprocal social interactions, in *Shank3B* Cohort 2.
**Figure S17**. CX546 did not reduce the high levels of repetitive self‐grooming in *Shank3B* in an empty cage.
**Figure S18**. CX546 did not affect open field exploratory locomotion.
**Figure S19**. 7,8‐DHF did not improve the parameters of male–female reciprocal social interaction which were lower in *Shank3B*.
**Figure S20**. 7,8‐DHF showed a trend toward reducing the high levels of self‐grooming in male *Shank3B*.
**Figure S21**. 7,8‐DHF had no effect on open field exploratory locomotion in *Shank3B*.
