# Supplementary material for: Hypothesis‐driven investigations of diverse pharmacological targets in two mouse models of autism
Source: Autism Res. 2019 Jan 17;12(3):401–21. doi: 10.1002/aur.2066 (PMC6402976; doi:10.1002/aur.2066)
Supplement: Supplementary file 1 — Figure S1. The GABA‐A agonist gaboxadol had no significant effects on three‐chambered social approach in BTBR mice. Figure S2. The GABA‐A agonist gaboxadol increased only one parameter of male–female reciprocal social interactions in BTBR. Figure S3. Gaboxadol had no significant effects on elevated plus‐maze, Cohort 1. Figure S4. Gaboxadol had no significant effects on elevated plus‐maze, Cohort 2. Figure S5. Gaboxadol had no significant effects on light↔dark transitions. Figure S6. The TrkB receptor agonist 7,8‐dihydroxyflavone (7,8‐DHF), had no effect on open field exploratory locomotion in BTBR. Figure S7. 7,8‐DHF did not reverse the social deficit in BTBR mice in male–female reciprocal social interactions. Figure S8. 7,8‐DHF did not reduce the high levels of repetitive self‐grooming in BTBR. Figure S9. The mTOR inhibitor rapamycin did not reduce the high levels of repetitive self‐grooming in BTBR. Figure S10. Rapamycin did not affect three‐chambered social approach. Figure S11. Gaboxadol had no effect on parameters of male–female reciprocal social interactions. Figure S12. Rapamycin had no consistent effects on open field exploratory locomotor behavior. Figure S13. D‐cycloserine, an agonist of the glycine‐B site on the NMDA receptor, 320 mg/kg, increased parameters of male–female reciprocal social interaction in Shank3B null mutant mice. Figure S14. D‐cycloserine 320 mg/kg induced hyperlocomotion. Figure S15. D‐cycloserine reduced the high levels of self‐grooming in Shank3B mice only at the high dose which induced hyperlocomotion. Figure S16. Ampakine CX546 had no effect on parameters of social and self‐grooming behaviors during male–female reciprocal social interactions, in Shank3B Cohort 2. Figure S17. CX546 did not reduce the high levels of repetitive self‐grooming in Shank3B in an empty cage. Figure S18. CX546 did not affect open field exploratory locomotion. Figure S19. 7,8‐DHF did not improve the parameters of male–female reciprocal social interaction whic [file AUR-12-401-s001.docx]

**Supplementary Figure S1**

**Gaboxadol**

**3-Chambered Social Approach**

**B6**

**BTBR**

**A**

**B**


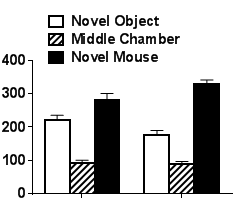


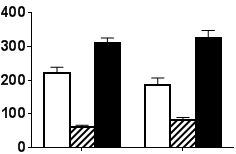


******

******

******

*****

**Chamber Time (sec)**

**Gab**

**N=11**

**Gab**

**N=14**

**Veh**

**N=15**

**C**

**D**


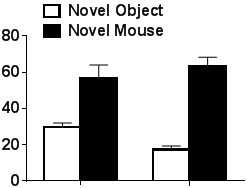


******


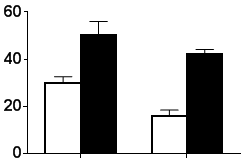


******

**Chamber Time (sec)**

******

******

**Sniffing Time (sec)**

**Sniffing Time (sec)**

**Veh**

**N=15**

**Gab**

**N=11**

**Gab**

**N=14**

**Veh**

**N=13**

**E**

**F**


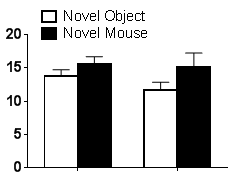

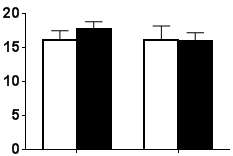


**Veh**

**N=13**

**Total Entries**

**Total Entries**

**Gab**

**N=11**

**Gab**

**N=14**

**Veh**

**N=15**

**Veh**

**N=13**

Supplementary Figure S1. The GABA-A agonist gaboxadol had no significant effects on 3-chambered social approach in either B6 or BTBR mice, Cohort 3. All groups displayed normal sociability, spending more time in the chamber with the novel mouse than in the chamber with the novel object, and spending more time sniffing the novel mouse than sniffing the novel object. *Note that this cohort of BTBR did not display its usual absence of sociability in this assay*. No differences were detected in entries into the side chambers, the internal control for general exploratory activity. A) B6 chamber time (One-Way ANOVA F_5, 62_ = 0.504, p<0.001; vehicle: *p<0.05, gaboxadol 3 mg/kg: **p<0.01; gaboxadol 5 mg/kg: **p<0.01; (B) BTBR chamber time (F_5, 28_ = 0.2451, p<0.001), vehicle: **p<0.01; gaboxadol 3 mg/kg: *p<0.05; gaboxadol 5 mg/kg: **p<0.01; C) B6 sniff time, vehicle: t_1,8_ = 4.08, **p<0.01; gaboxadol 3 mg/kg: t_1,10_ = 2.77, **p<0.01; gaboxadol 5 mg/kg: t_1,10_ = 4.64, **p<0.001; D) BTBR sniff time, vehicle: t_1,20_ = 3.53, **p<0.001; gaboxadol 3 mg/kg: t_1,20_ = 5.61, **p<0.001; gaboxadol 5 mg/kg: t_1,22_ = 4.30, **p<0.001; (E) B6 entries into side chambers, vehicle: t_1,8_ = 1.123, NS; gaboxadol 3 mg/kg: t_1,10_ = 0.53, NS; gaboxadol 5 mg/kg: t_1,10_ = 0.268, NS; (F) BTBR entries into side chambers, vehicle: t_1,20_ = 0.356, NS; gaboxadol 3 mg/kg: t_1,20_ = 1.81, NS; gaboxadol 5 mg/kg: t_1,22_ = 0.441, NS.

**Supplementary Figure S2**

**Gaboxadol Cohort 1**

**Male Female Reciprocal Social Interaction**

**A**

**B**


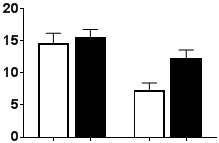

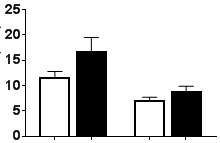


*****

*****

**Nose-to-Nose**

**Duration (sec)**

**Nose-to-Nose**

**(# Bouts)**

**Gab**

**N=6**

**Gab**

**N=6**

**Gab**

# N=6

**Gab**

**N=6**

**Veh**

**N=7**

**Veh**

**N=8**

**Veh**

**N=7**

**Veh**

**N=8**

**BTBR**

**BTBR**

**B6**

**B6**

**C**

**D**


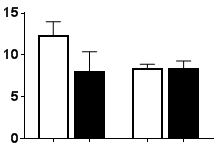


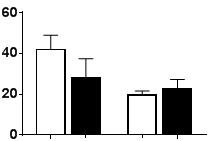


**Nose-to-Anogenital**

**Duration (sec)**

**Nose-to-Anogenital**

**(# Bouts)**

**Gab**

**N=6**

**Gab**

**N=6**

**Gab**

**N=6**

**Gab**

**N=6**

**Veh**

**N=7**

**Veh**

**N=8**

**Veh**

**N=7**

**Veh**

**N=8**

**BTBR**

**BTBR**

**B6**

**B6**

**E**

**F**


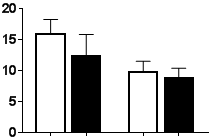

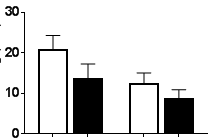


**Following (sec)**

**Following
(# bouts)**

**BTBR**

**BTBR**

**B6**

**B6**

**Gab**

**N=6**

**Gab**

**N=6**

**Gab**

**N=6**

**Gab**

**N=6**

**Veh**

**N=7**

**Veh**

**N=8**

**Veh**

**N=7**

**Veh**

**N=8**

Supplementary Figure S2. Gaboxadol, 5 mg/kg, increased only one parameter of male-female reciprocal social interactions in B6 and BTBR mice, Cohort 1. A) Time spent in nose-to-nose sniffing (Two-Way ANOVA, genotype: F_1,23_ = 15.4, p<.001, treatment: F_1,23_ = 4.60, p<.05); B) Bouts of nose-to-nose sniffing (genotype: F_1,23_ = 12.9, p<0.001, treatment: F_1,23_ = 4.09, p = 0.055); C) Time spent in nose-to-anogenital sniffing (genotype: F_1,23_ = 4.72, p<0.05, treatment: F_1,23_ = 0.731, NS); D) Bouts of nose-to-anogenital sniffing (genotype: F_1,23_ = 1.32, NS, treatment: F_1,23_ = 1.77, NS); E) Time spent following (genotype: F_1,23_ = 4.14, p=0.054, treatment: F_1,23_ = 2.76, NS); F) Bouts of following (genotype: F_1, 23_ = 3.90, p<.060, treatment: F_1, 23_ = 0.818, NS).

**Supplementary Figure S3**

**Gaboxadol, 3 mg/kg**

**Elevated Plus-Maze**

**Cohort 1**

**A**

**B**

**C**

Supplementary Figure S3. Gaboxadol, 3 mg/kg, had no significant effects on the elevated plus-maze test for anxiety-related behaviors in either B6 or BTBR mice, Cohort 1. A) Time in open arms (Two-Way ANOVA strain: F_1, 51_ = 18.59, p<.001, treatment: F_1, 51_ = 0.567, NS); (B) Number of entries into the open arms (strain: F_1, 51_ = 12.2, p<.001, treatment: F_1, 51_ = 0.080, NS); (C) Total entries into all four arms, an internal measure of general activity (strain: F_1, 51_ = 2.147, NS, treatment: F_1, 51_ = 0.0362, NS).

**Supplementary Figure S4**

**Gaboxadol 3 mg/kg**

**Elevated Plus-Maze**

**Cohort 2**

**A**

**B**

**C**

Supplementary Figure S4. Gaboxadol, 3 mg/kg, had no significant effects on the elevated plus-maze test for anxiety related behaviors in either B6 or BTBR mice, Cohort 2. A) Time in open arms (strain: F1, 34 = 12.49, p<.01, treatment: F1, 34 = 1.49, NS). B) Number of entries in the open arms (strain: F1, 34 = 5.66, p<.05, treatment: F1, 34 = 1.27, NS). C) Total entries into all four arms (strain: F1, 34 = 1.169, NS; treatment: F1, 34 = 0.0794, NS).

**Supplementary Figure S5**

**Gaboxadol**

**Light↔Dark Transitions**

**5mg/kg**

*****

**A**

**B**

**3mg/kg**

**C**

**D**

Supplementary Figure S5. Gaboxadol, administered acutely at two doses, generally had no anxiolytic or anxiogenic effects in either B6 or BTBR mice in the light↔dark exploration test for anxiety-related behaviors. A) Time spent in the larger, open, brightly lit chamber, vehicle versus 5 mg/kg (Two-Way ANOVA strain: F_1,50_ = 8.517, p<0.01, treatment: F_1,50_ = 0.332, NS); B) Number of transitions between compartments, vehicle versus 5 mg/kg (strain: F_1,50_ = 1.165, NS, treatment: F_1,50_ = 1.21, NS). C) Time spent in light chamber, vehicle versus 3 mg/kg (strain: F_1,34_ = 0.258, NS, treatment: F_1, 34_ = 1.92, NS); D) Number of transitions between compartments, vehicle versus 3 mg/kg (strain: F_1,34_ = 0.385, NS, treatment: F_1,34_ = 2.50, NS).

**Supplementary Figure S6**

**7,8-DHF**

**Open Field Activity**

**B6**

**BTBR**

**A**

**B**


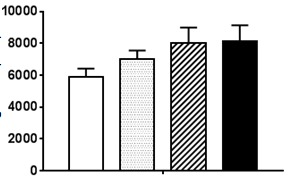

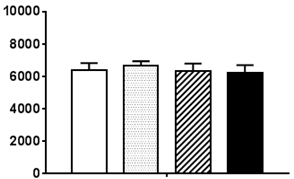


**Total distance travelled**

**Total distance travelled**

**7.5**

**N=15**

**2.5**

**N=13**

**7.5**

**N=15**

**5**

**N=14**

**Veh**

**N=14**

**5**

**N=14**

**2.5**

**N=13**

**Veh**

**N=14**

**mg/kg i.p.**

**mg/kg i.p.**

**mg/kg i.p.**

Supplementary Figure S6. The TrkB receptor agonist 7,8-dihydroxyflavone (7,8-DHF), had no effect on open field exploratory locomotion, during a 60 minute test session under 40 lux lighting conditions, in either B6 or BTBR mice. No genotype difference was detected (Two-Way ANOVA: genotype: F_1,108_ = 3.3, NS). A) B6 total distance traveled showed no differences between vehicle, 2.5 mg/kg, 5.0 mg/kg, and 7.5 mg/kg treatment groups (F_3,44_= 0.0272, NS). B) BTBR total distance traveled showed no differences between vehicle, 2.5 mg/kg 5.0 mg/kg, and 7.5 mg/kg treatment groups (F_3,44_= 0.31, NS). These open field results corroborate the normal side chamber entry scores in the 3-chambered social approach assay, supporting the interpretation that 7,8-DHF improved 3-chambered social approach in BTBR mice at doses that did not affect general exploratory activity. No sex differences were detected.

**Supplementary Figure S7**

**7,8-DHF**

**Male-Female Social Interactions**

**BTBR**

**B6**

**G**

**H**

**E**

**F**

**C**

**D**

**A**

**B**

**I**

**J**

**M**

**N**

**K**

**L**

**I**

**J**

B6

BTBR

**K**

**L**

**M**

**N**

**I**

**J**

**K**

**L**

**M**

**N**

**O**

**P**

**R**

**Q**

Supplementary Figure S7. 7,8-DHF did not affect the social deficit in BTBR mice on parameters of male-female reciprocal social interactions, and had no deleterious effects on normal male-female social interactions in B6 control mice. The 7.5 mg/kg dose (which improved 3-chambered social approach in BTBR as shown in Figure 2) did not increase the (A,B) lower scores of BTBR as compared to B6 on parameters of time spent engaged in nose-to-nose sniffing (genotype: F_1,36_ = 16.6, p<0.001, treatment: F_1,36_ = 0.498, NS); (C,D) Time spent engaged in nose-to-anogenital sniffing (genotype: F_1,36_ = 7.13, p<0.05, treatment: F_1,36_ = 0.256, NS); (E,F) Number of approaches by the subject male to the front of the estrous female (genotype: F_1,36_ = 2.70, NS, treatment: F_1,36_ = 0.1208, NS); or (G,H) Time spent by the subject male in following the estrous female (genotype: F_1,36_ = 3.89, NS, treatment: F_1,36_ = 0.301, NS). Additional parameters scored for the subject males during the interaction session were similarly unaffected by 7,8-DHF at 7.5 mg/kg, including (I,J) Arena exploration (genotype: F_1,36_ = 28.7, p<0.001, treatment: F_1,36_ = 1.076, NS); (K,L) Time spent self-grooming (genotype: F_1,36_ = 6.14, p<0.05, treatment: F_1,36_ = 0.871, NS); (M,N) Time spent digging in the litter (genotype: F_1,36_ = 49.0, p <0.001, treatment: F_1,36_ = 3.05, NS); (O,P) Number of episodes of pushing and crawling past each other (genotype: F_1,36_ = 0.631, NS, treatment: F_1,36_ = 0.881, NS); and (Q,R) Number of ultrasonic vocalizations (genotype: F_1,36_ = 3.95, p = 0.0545, NS, treatment: F_1,36_ = 0.101, NS).

**Supplementary Figure S8**

**7,8-DHF**

**Self-Grooming**

**A**

**B**

**BTBR**

**B6**


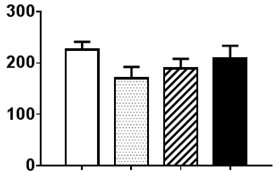

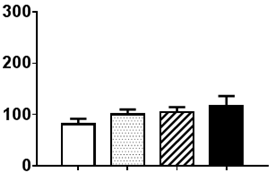


**Grooming Time (sec)**

**Grooming Time (sec)**

**2.5**

**N=15**

**Veh**

**N=15**

**5**

**N=15**

**7.5**

**N=15**

**5**

**N=15**

**7.5**

**N=15**

**2.5**

**N=15**

**Veh**

**N=15**

**mg/kg i.p.**

**mg/kg i.p.**

Supplementary Figure S8. 7,8-DHF did not affect the characteristic high levels of repetitive self-grooming in BTBR mice, and had no effect on the lower levels of grooming in B6 control mice (Two Way ANOVA, strain: F_1,111_ = 62.1, p<0.001; treatment: F_3,11_ = 0.812, NS). No sex differences were detected.

**Supplementary Figure S9**

**Rapamycin**

**Self-Grooming**

**Cohort 2**

**Cohort 1**

**A**

**B**

Supplementary Figure S9. The mTOR inhibitor rapamycin showed no significant effects on spontaneous repetitive self-grooming behavior in B6 and BTBR mice at 10 mg/kg i.p., administered semi-chronically. A) Cohort 1. Rapamycin treatment had no effect on normal levels of self-grooming in B6, but showed a trend for decreasing the high levels of self-grooming in BTBR mice (strain: F_1,39_ = 13.24, p<0.001, treatment: F_1,39_ = 3.634, p = 0.069). B) Cohort 2. Rapamycin had no effect on self-grooming in either B6 or BTBR (strain: F_1,34_ = 0.478, NS, treatment: F_1,34_ = 0.050, NS). Cohort 2 BTBR did not display the high level of self-grooming usually seen in this inbred strain.

**Supplementary Figure S10**

**Rapamycin**

**3-Chambered Social Approach**

**BTBR**

**B6**

**A**

**B**

******

******

******

******

******

******

**C**

**D**

******

******

******

******

**E**

**F**

Supplementary Figure S10. Rapamycin, 10 mg/kg, had no effect on 3-chambered social approach in B6 and BTBR mice. All groups displayed normal sociability, spending more time in the chamber with the novel mouse than in the chamber with the novel object, and spending more time sniffing the novel mouse than sniffing the novel object. *Note that this cohort of BTBR did not display its usual absence of sociability in this assay*. No differences were detected in entries into the side chambers, the internal control for normal exploratory activity. A) B6 chamber time (One-Way ANOVA F_3, 36_ = 0.0921, **p<0.001. Tukey’s multiple comparisons post hoc: vehicle novel mouse versus novel object p<0.05, rapamycin novel mouse versus novel object **p<0.01). (B) BTBR chamber time (F_3, 38_ = 0.942, **p<0.001, vehicle novel mouse vs. novel object **p<0.01, rapamycin novel mouse versus novel object **p<0.01). C) B6 sniff time (vehicle t_1,20_ = 3.369, **p<0.001, rapamycin t_1,16_ = 5.542, **p<0.001). (D) BTBR sniff time (vehicle t_1,20_ = 3.786, **p<0.01, rapamycin t_1,18_ = 3.38, **p<0.01). E) B6 entries (vehicle t_1,20_ = 0.615, NS, rapamycin t_1,16_ = 1.602, NS). F) BTBR entries (vehicle t_1,18_ = 0.224, NS, rapamycin t_1,18_ = 0.558, NS).

**Supplementary Figure S11**

**Rapamycin**

**Male Female Reciprocal Social Interactions**

**A**

**B**

**C**

**D**

**E**

**F**

Supplementary Figure S11. Rapamycin, 10 mg/kg i.p., had no effect on parameters of male-female reciprocal social interactions in B6 and BTBR mice. A) Time spent in nose-to-nose sniffing (Two-Way ANOVA, genotype: F_1,23_ = 15.4, p<.001, treatment: F_1,23_ = 4.60, p<.05, Tukey’s multiple comparisons detected no significant differences between vehicle and drug within genotype); B) Bouts of nose-to-nose sniffing (genotype: F_1,23_ = 12.9, p<0.001, treatment: F_1,23_ = 4.09, p=0.055, non-significant trend); C) Time spent in nose-to-anogenital sniffing (genotype: F_1,23_ = 4.72, p<0.05, treatment: F_1,23_ = 0.731, NS); D) Bouts of nose-to-anogenital sniffing (genotype: F_1,23_ = 1.32, NS, treatment: F_1,23_ = 1.77, NS); E) Time spent following (genotype: F_1,23_ = 4.14, p=0.054, treatment: F_1,23_ = 2.76, NS); F) Bouts of following (genotype: F_1, 23_ = 3.90, p<.060, treatment: F_1, 23_ = 0.818, NS).

**Supplementary Figure S12**

**Rapamycin**

**Open Field Activity**

**Cohort 2**

**Cohort 1**

**A**

**B**

Supplementary Figure S12. Rapamycin, 10 mg/kg, had minimal effects on open field exploratory locomotor behavior in B6 and BTBR mice. A) Cohort 1. Rapamycin had no effect in B6, but increased total distance traveled in BTBR mice (strain: F_1,38_ = 5.60, p<0.05, treatment: F_1,38_ = 7.402, p<0.01, Tukey’s multiple comparisons BTBR vehicle versus rapamycin *p<.05). B) Cohort 2. Rapamycin had no effect on open field activity in either B6 or BTBR (strain: F_1,21_ = 0.835, NS, treatment: F_1,21_ = 1.076, NS). Although small Ns were employed, no trend toward higher open field activity was detected in Cohort 2 BTBR treated with rapamycin.

**Supplementary Figure S13**

**D-cycloserine 320mg/kg**

**Male Female Reciprocal Social Interaction**

**A**

**B**

**C**

**D**

*****

******

******

******

******

*****

*****

**E**

**F**

******

******

**G**

**I**

**H**

Supplementary Figure S13. D-cycloserine, an agonist of the glycine-B site on the NMDA receptor, at an acute dose of 320 mg/kg, increased parameters of male-female reciprocal social interaction in WT and *Shank3B* null mutant mice. *Note that this dose dramatically increased general activity, as shown in Supplementary Figure S14*. A) Time spent in nose-to-nose sniffing (Two-Way ANOVA genotype: F_1,50_ = 15.44, p<0.001, treatment: F_1,50_ = 1,54 = 16.93, p<0.001, Tukey’s multiple comparisons **p<.01 vehicle versus d-cycloserine in both WT and *Shank3B*); B) Bouts of nose-to-nose sniffing (genotype: F_1,54_ = 13.51, p<0.001, treatment: F_1,54_ = 16.75, p<0.001, **p<.01 for WT, *p<.05 for *Shank3B*); C) Time spent in nose-to-anogenital sniffing (genotype: F_1,54_ = 0.4068, NS, treatment: F_1,54_ = 4.089, p<.05, Tukey’s *p<.05 for *Shank3B*). D) Bouts of nose-to-anogenital sniffing (genotype: F_1,54_ = 1.409, NS, treatment: F_1,54_ = 1,54 = 13.95, p<0.001, *p<.05 WT, **p<.01 *Shank3B*); E) Time spent following (genotype: F_1,54_ = 11.92, p<.01, treatment: F_1,54_ = 5.597, **p<0.01 *Shank3B*). F) Bouts of following (genotype: F_1, 54_ = 1.5, p=0.0226, treatment: F_1,54_ = 11.9, p<0.01, **p<.01 *Shank3B*). G) Number of approaches (genotype: F_1,54_ = 3.649, non-significant trend, p = 0.061, treatment: F_1,54_ = 1.072, p=.305, NS). H) Ultrasonic vocalizations shown in one minute time bins (treatment: F_3, 54_ = 1.545, NS) and I) summed across the five minute test session (Genotype: F_1,54_ = 0.1502, NS, treatment: F_1,54_ = 4.327, p = 0.0423, Tukey’s multiple comparisons detected no significance for treatment within genotype, likely due to variability in vehicle treated groups and scores of zero in groups treated with d-cycloserine 320 mg/kg).

**Supplementary Figure S14**

**D-cycloserine**

**Open Field Activity**

**32 mg/kg**

**320 mg/kg**

******

******

**A**

**B**

Supplementary Figure S14. D-cycloserine induced dramatic hyperlocomotion in both WT and *Shank3B* mice, when administered acutely at the high dose of 320 mg/kg, but not at the lower dose of 32 mg/kg, as measured in a 30 minute open field arena. A) 32 mg/kg (genotype: F_1,105_ = 13.95, p<.001, treatment: F_1,105_ = 2.46, NS). B) 320 mg/kg (genotype: F_1,107_  = 0.001, NS; treatment: F_1,107_ = 279.3, p<<0.001, Tukey’s posthoc significant for both WT **p<.0001 and *Shank3B* **p<.0001. No sex differences were detected (F_1,1_ = 0.329, p=.0568).

**Supplementary Figure S15**

**D-cycloserine**

**Self-Grooming**

**320 mg/kg**

**32 mg/kg**

**Grooming time (sec)**

**Grooming time (sec)**

******

******

**A**

**B**

Supplementary Figure S15. D-cycloserine reduced the high levels of self-grooming in *Shank3B* mice only at the high dose. A) 32 mg/kg i.p. (Two-Way ANOVA genotype: F_1,102_ = 27.02, p<.001, treatment F_1,102_ = 1.338, NS). B) 320 mg/kg i.p. (Two-Way ANOVA genotype: F_1,105_ = 17.08, p<.001, treatment F_1,105_ = 45.68, p<.001; Tukey’s posthoc comparison **p<.01 for vehicle versus d-cycloserine 320 mg/kg in both WT and *Shank3B*). Sex differences were detected (F_3,101_ = 6.365, p< .001), with *Shank3B* males displaying higher levels of grooming than *Shank3B* females in the groups shown in panel B. Note that the 320 mg/kg dose of d-cycloserine produced high scores on open field locomotion, indicating that hyperactivity may have competed with self-grooming.

**Supplementary Figure S16**

**CX546 15 mg/kg**

**Male Female Reciprocal Social Interaction**

**Cohort 2**

**A**

**B**

**C**

**D**

**E**

**F**

**G**

**H**

**I**

**J**

**J**

**K**

*****

*****

Supplementary Figure S16. The ampakine CX546, a positive allosteric modulator glutamatergic AMPA receptor, 15 mg/kg i.p., had no effect in either WT or *Shank3B* males of Cohort 2 on parameters of social and self-grooming behaviors during male-female reciprocal social interactions. *Note that Ns were low in Cohort 2*. (A) Time spent by the male subject mouse in nose-to-nose sniffing of an estrous female (Two-Way ANOVA genotype: F_1,27_ = 0.0165, NS, treatment: F_1,27_ = 0.508, NS); (B) number of bouts of nose-to-nose sniffing (genotype: F_1,27_ = 0.450, NS, treatment: F_1,27_ = 0.161, NS); C) nose-to-nose sniffing time (genotype: F_1,27_ = 9.823, p<.01, treatment: F_1,27_ = 0.049, NS); D) Number of bouts of nose-to-nose sniffing (genotype: F_1,27_ = 1.527, NS, treatment: F_1,27_ = 1.32, NS); E) following (genotype: F_1,27_ = 4.03, p=.055, treatment: F_1,27_ = 0.114, NS); F) Number of bouts of following (genotype: F_1,27_ = 9.21, p<.01, treatment: F_1,27_ = 0.0088, NS); G) Time spent grooming during the male-female social interaction test (genotype: F_1,27_ = 11.04, p<.01, treatment: F_1,27_ = 0.937, NS); H) Number of grooming bouts (genotype: F_1,27_ = 0.028, p<.05, treatment: F_1,27_ = 1.80, NS); I) Total number of approaches (genotypes: F_1,27_ = 0.904, NS, treatment: F_1,27_ = 0.107, NS). J) Ultrasonic vocalizations were unexpectedly reduced by CX546 treatment, expressed in one minute time bins (time: F_4,230_ = 4.223, genotype: p<0.01, treatment: F_3, 230_ = 17.17, p<0.001), and K) summed across the five minute test session (genotype: F_1,46_ = 0.0327, NS, treatment: F_1, 46_ = 13.6, p<0.001, Tukey’s multiple comparisons WT *p<.05, *Shank3B* *p<.05).

**Supplementary Figure S17**

**CX546**

**Self-Grooming**

**Cohort 2**

**Cohort 1**

**A**

**B**

Supplementary Figure S17. CX546 15 mg/kg did not reduce the high levels of repetitive self-grooming which characterize *Shank3B* mice. WT and *Shank3B* were individually placed in an empty cage for a 10 minute habituation session, followed by a 10 minute test session which was scored from coded videos by a rater uninformed of genotype and treatment. A) Cohort 1, CX546 (Two-Way ANOVA, genotype: F_1,91_ = 17.17, p<0.001, treatment: F_1,91_ = 3.563, NS). B) Cohort 2, CX546 (genotype: F_1,93_ = 16.29, p<0.001; treatment F_1,93_ = 2.896, NS). Sex differences were detected in both Cohort 1 (F_3,87_ = 10.65, p<0.01) and Cohort 2 (F_1,89_ = 7.104, p<0.01), with males showing overall higher grooming scores than females.

**Supplementary Figure S18**

**CX456**

**Open Field Activity**

**Cohort 2**

**Cohort 1**

**A**

**B**

**A**

Supplementary Figure S18. CX546 15 mg/kg did not affect open field exploratory activity in WT or *Shank3B* mice in either Cohort 1 or Cohort 2. (A) Cohort 1 (Two-Way ANOVA genotype: F_1,95_ = 31.96, p<0.001, treatment: F_1,95_ = 3.839, NS); (B) Cohort 2 (genotype: F_1,95_ = 41.89, p<.001, treatment: F_1,95_ = 0.784, NS). Sex differences were not detected in Cohort 1 (F_1,95_ = 0.337, NS), but were significant in Cohort 2 (F_1,95_ = 4.10, p<.05), in which the open field locomotion was lower in females than in males.

**Supplementary Figure S19**

**7,8-DHF**

**Male Female Reciprocal Social Interaction**

**A**

**B**

**C**

**D**

**E**

**F**

**I**

**G**

**H**

**J**

**K**

Supplementary Figure S19. 7,8-DHF, 5 mg/kg i.p., had no significant effects on parameters of male-female reciprocal social interaction, including parameters that were lower in *Shank3B* than WT mice. A) Time spent in nose-to-nose sniffing (Two-Way ANOVA, genotype: F_1,56_ = 0.0032, NS, treatment F_1,56_ = 1.001, NS); B) Bouts of nose-to-nose sniffing (genotype: F_1,56_ = 0.540, NS, treatment: F_1,56_ = 0.030, NS); C) Time spent in nose-to-anogenital sniffing (genotype: F_1,56_ = 9.761, p<.01, treatment: F_1,56_ = 0.122, NS); D) Bouts of nose-to-anogenital sniffing (genotype: F_1,56_ = 1.728, NS, treatment: F_1,56_ = 1.095, NS); E) Time spent following (genotype: F_1,56_ = 0.276, NS, treatment: F_1,56_ = 0.664, NS); F) Bouts of following (genotype: F_1, 56_ = 5.872, p<.05, treatment: F_1, 56_ = 0.335, NS); G) Time spent self-grooming during the male-female social interactions session (genotype: F_1,56_ = 2.172, NS, treatment: F_1,56_ = 0.591, NS); H) Number of bouts of self-grooming (genotype: F_1, 56_ = 5.985, p<.05, treatment: F_1, 56_ = 0.0015, NS); I) Number of approaches (genotype: F_1,56_ = 0.420, NS, treatment: F_1,56_ = 0.420, NS); J) Ultrasonic vocalizations displayed in 1 minute time bins; K) Ultrasonic vocalizations summed for 5 minute test session (genotype: F_1,56_ = 11.59, p< 0.01, treatment: F_1,56_ = 0.3554, NS).

**Supplementary Figure S20**

**7,8-DHF**

**Self-Grooming**

**Female**

**Male**

**A**

**B**

**p = 0.053**

**A**

Supplementary Figure S20. 7,8-DHF, 5 mg/kg, did not significantly affect self-grooming in WT and *Shank3B* mice, but showed a trend toward reducing the high levels of self-grooming in male *Shank3B* mice. A) Male *Shank3B* displayed more repetitive grooming during the 10 minute test session in an empty cage than WT (Two-Way ANOVA genotype: F_1,55_ = 7.72, p<.01). 7,8-DHF treatment produced a trend toward less self-grooming in *Shank3B* males (treatment: F_1,55_ = 3.91, p = 0.053). B) Female *Shank3B* displayed more repetitive self-grooming than WT; 7,8-DHF had no effect (genotype: F_1,56_ = 11.95, p<0.01, treatment: F_1,56_ = 0.263, NS).

**Supplementary Figure S21**

**7,8-DHF**

**Open Field Activity**

**Male**

**Female**

**A**

**B**

**A**

Supplementary Figure S21. 7,8-DHF, 5 mg/kg, had no effect on open field exploratory locomotion in male or female WT or *Shank3B*. A) Females (genotype: F_1,57_ = 7.852, p<0.001, treatment: F_1,57_ = 4.128, p<0.05; Tukey’s posthoc multiple comparisons NS for vehicle versus 7,8-DHF within genotype, significance was only for the comparison of WT+vehicle versus *Shank3B*+7,8-DHF). B) Males (genotype: F_1,57_ = 4.61, p<0.05, treatment: F_1,57_ = 1.16, NS). No significant sex differences were detected (F_1,118_ = 0.026, NS).
